# Antibiotic-modulated microbiome suppresses lethal inflammation and prolongs lifespan in Treg-deficient mice

**DOI:** 10.1186/s40168-019-0751-1

**Published:** 2019-11-07

**Authors:** Baokun He, Yuying Liu, Thomas K. Hoang, Xiangjun Tian, Christopher M. Taylor, Meng Luo, Dat Q. Tran, Nina Tatevian, J. Marc Rhoads

**Affiliations:** 1 Department of Gastroenterology, Shanghai General Hospital, Shanghai Jiao Tong University School of Medicine, Shanghai, 200080 China; 2 Shanghai Key Laboratory of Pancreatic Disease, Shanghai General Hospital, Shanghai Jiao Tong University School of Medicine, Shanghai, 201620 China; 3 Division of Gastroenterology, Department of Pediatrics, The University of Texas Health Science Center at Houston McGovern Medical School, Houston, TX 77030 USA; 4 Department of Bioinformatics & Computational Biology, The University of Texas MD Anderson Cancer Center, Houston, TX 77030 USA; 5 Department of Microbiology, Immunology & Parasitology, Louisiana State University School of Medicine, Children’s Hospital, New Orleans, Louisiana, 70118 USA; 6 Department of Pathology and Laboratory Medicine, The University of Texas Health Science Center at Houston McGovern Medical School, Houston, TX 77030 USA

**Keywords:** Treg deficiency, IPEX syndrome, Lethal inflammation, Gut microbiota, Bile acid, IL-6

## Abstract

**Background:**

Regulatory T cell (Treg) deficiency leads to IPEX syndrome, a lethal autoimmune disease, in Human and mice. Dysbiosis of the gut microbiota in Treg-deficient scurfy (SF) mice has been described, but to date, the role of the gut microbiota remains to be determined.

**Results:**

To examine how antibiotic-modified microbiota can inhibit Treg deficiency-induced lethal inflammation in SF mice, Treg-deficient SF mice were treated with three different antibiotics. Different antibiotics resulted in distinct microbiota and metabolome changes and led to varied efficacy in prolonging lifespan and reducing inflammation in the liver and lung. Moreover, antibiotics altered plasma levels of several cytokines, especially IL-6. By analyzing gut microbiota and metabolome, we determined the microbial and metabolomic signatures which were associated with the antibiotics. Remarkably, antibiotic treatments restored the levels of several primary and secondary bile acids, which significantly reduced IL-6 expression in RAW macrophages in vitro. IL-6 blockade prolonged lifespan and inhibited inflammation in the liver and lung. By using IL-6 knockout mice, we further identified that IL-6 deletion provided a significant portion of the protection against inflammation induced by Treg dysfunction.

**Conclusion:**

Our results show that three antibiotics differentially prolong survival and inhibit lethal inflammation in association with a microbiota—IL-6 axis. This pathway presents a potential avenue for treating Treg deficiency-mediated autoimmune disorders.

## Background

Mutations or deletions of the forkhead box protein 3 (Foxp3) gene, which encodes a major transcription factor required for regulatory T (Treg) cell development and function, result in Treg deficiency in both human and mouse [1–4]. Treg deficiency causes the immunodysregulation polyendocrinopathy enteropathy syndrome with X-linked inheritance (IPEX syndrome), which is an autoimmune disease associated with eczema, severe enteropathy, type I diabetes, thyroiditis, hemolytic anemia, and thrombocytopenia in children [1, 3]. The scurfy (SF) mouse with the same Foxp3 mutation displays a similar phenotype with multi-organ inflammation, early-onset dermatitis, and rapid death due to a lymphoproliferative syndrome induced by Treg deficiency [2, 4]. Moreover, mutations of several other genes, including *LRBA*, *STAT5B*, *IL2RA*, *STAT1*, *STAT3*, *CTLA4*, *ITCH*, and *DOCK8*, lead to IPEX-like syndromes by disrupting Treg cells [5–7]. To date, IPEX syndrome and IPEX-like syndrome still pose a significant therapeutic challenge. Treatment of infants diagnosed with IPEX syndrome using immunosuppressive drugs may transiently reduce clinical manifestations but is largely unsuccessful [8]. At the present time, potentially curative therapy relies on the transplantation of hematopoietic stem cells, but this procedure is limited by donor availability and a high risk-benefit ratio [9].

Recent studies indicate that gut microbial dysbiosis is critically linked to the pathophysiology of autoimmune diseases, including inflammatory bowel disease, autoimmune arthritis, type I diabetes, and multiple sclerosis [10, 11]. Moreover, our previous studies have demonstrated the role of gut microbiota in the development of lethal inflammation induced by Treg-deficiency in SF mice [12]. Strategies that modulate the gut microbiota have been identified as a potential avenue to prevent and treat autoimmune diseases. Diet, antibiotics, and probiotics represent feasible approaches that affect host immunity by altering the gut microbiota [12–14]. Some previous studies have revealed that modulation of the gut microbiota by antibiotics may inhibit autoimmunity and reduce inflammation in autoimmune disease models [15, 16]. Additionally, our findings indicate that a probiotic, *Lactobacillus reuteri* (*L. reuteri*), prolongs lifespan and inhibits autoimmunity in SF mice [12]. However, it remains unclear to what extent these microbial population changes and what mechanisms are involved in the immunosuppressive benefits in individuals with Treg dysfunction.

In the present study, we show that Treg-deficient SF mice, when treated with antibiotics, had prolonged survival and reduced multi-organ inflammation. Moreover, antibiotic treatment altered the gut microbiota and metabolome in SF mice. Further experiments showed that IL-6 played a critical role in the development of lethal inflammation induced by Treg deficiency.

## Methods

### Animals

Wild-type (WT) C57BL/6, B6.129S2-IL6^tm1Kopf^/J, and heterozygous B6.Cg-Foxp3^sf^/J mice were purchased from Jackson Laboratories and allowed to acclimatize for 2 weeks before experimentation. Scurfy (SF) mice with hemizygous B6.Cg-Foxp3^sf^/Y was generated by breeding heterozygous B6.Cg-Foxp3^sf^/J female to C57BL/6J male mice. All mice were housed in the animal facility at UT Health Science Center at Houston. All experimental procedures were approved by the IACUC (protocol number: AWC-17-0045).

### Antibiotic treatments of WT and SF mice

**Fig. 1:**
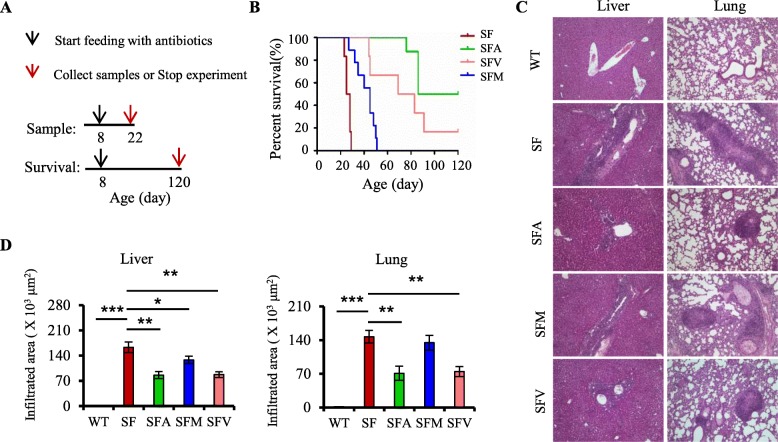
Antibiotics increase survival and inhibit inflammation in scurfy mice. **a** Scheme for antibiotic treatment on SF mice for sample analysis and survival observation. **b** Survival curves of SF mice with water (SF), ampicillin (SFA), metronidazole (SFM), or vancomycin (SFV) treatment (*n* = 6–10). **c** Representative H&E staining of liver and lung from WT, SF, SFA, SFM, and SFV mice (*n* = 6–9). **d** Quantitation of inflammatory infiltrates in the liver and lung of WT, SF, SFA, SFM, and SFV mice (*n* = 6–9). Data are presented as mean ± SEM. *p < 0.05, **p < 0.01, ***p < 0.001

SF or WT mice were fed with oral gavage of 150 mg/kg of ampicillin, 150 mg/kg of metronidazole, or 75 mg/kg of vancomycin, daily, from 8 to 21 days of age [17]. From 22 days of age to experimental closure, SF mice were treated with 1 g/L of ampicillin, 1 g/L of metronidazole, or 0.5 g/L of vancomycin in drinking water, respectively. Plasma and tissues were collected from WT or SF mice treated with water (SF), ampicillin (SFA), metronidazole (SFM), or vancomycin (SFV) at 22 days of age. For survival experiments, SF mice were given either water or antibiotics from 8 days of age to the date as indicated in Fig. 1a.

### IL-6 antibody treatment of SF mice

For determining the effect of IL-6 on autoimmunity in SF mice, 1 mg/kg of IL-6 antibody (MP520F3, Invitrogen, USA) (SF + αIL-6) or 1 mg/kg of IgG (Bio X Cell, USA) as control (SF + IgG) was intraperitoneally (i.p.) injected, once every 3 days, into SF mice from 8 days of age. Plasma and tissues were collected from SF + IgG and SF + αIL-6 mice at 22 days of age. For survival experiments, SF mice were given either IgG or IL-6 antibody from 8 days of age to the date as indicated in Fig. 5a.

### Stool microbial community analysis

Stool DNA was extracted by Quick Stool DNA Isolation Kit (Qiagen), according to the manufacturer’s protocol. The composition of the stool microbiota was analyzed by high-throughput sequencing analysis of 16S rRNA gene sequencing. Bacterial diversity, species composition, and abundance were assessed by QIIME-based microbiota analysis [18].

### Stool metabolome analysis

A total of 726 metabolites in stool were determined by a non-targeted metabolome platform including UPLC-MS/MS and GC-MS in Metabolon Inc. (USA). The metabolome data were analyzed by pattern recognition analyses (unsupervised principal component analysis and Hierarchical clustering) [17].

### CD25 antibody treatment of WT and IL-6^−/−^ mice

To determine the role of IL-6 in the development of inflammation induced by Treg depletion, WT and IL-6^−/−^ mice were given a daily i.p. injection of 150 mg/kg of CD25 antibody (Bio X Cell, USA) or IgG (Bio X Cell, USA). The antibodies were administered twice, at 8 to 9 days of age. Plasma and tissues were collected from WT and IL-6^−/−^ mice at 22 days of age.

### Histopathology

The liver and lung from different groups were fixed and processed by the Cellular and Molecular Morphology Core Lab (the Texas Medical Center Digestive Diseases Center, Houston, TX) and stained with hematoxylin and eosin (H&E). The area of lymphocyte infiltration of the liver and lung was independently measured by three people using Image J morphometry software (NIH, USA).

### Staining cells for flow cytometry analysis

Single-cell suspensions from the spleen were prepared by filtering the tissues through 40 μm cell strainers (BD Bioscience). For characterization of Treg cells, lymphocytes were surface-stained with fluorochrome-labeled CD4 and CD25 antibodies and intracellularly stained with the Foxp3 antibody (all from BioLegend). Intracellular staining was performed with a fixation/permeabilization kit, according to the manufacturer’s protocol (eBioscience). The data were collected from BD FACSCalibur and analyzed by FlowJo software (FlowJo, LLC).

### Multiplex cytokine assays and cell viability test

Plasma cytokine levels of IFN-γ, IL-2, IL-6, IL-4, IL-1β, TNFα, and IL-10 were examined using a mouse multi-spot proinflammatory panel kit from Meso Scale Discovery (MSD), according to the manufacturer’s protocol.

For determining the effect of bile acids on IL-6 expression and cell viability in RAW 264.7 murine macrophage cells, after 24 h from splitting 3000 cells into one well of 96-well plates, cells were pretreated with taurocholic acid sodium salt hydrate (Sigma), sodium tauroursodeoxycholate (Selleck), and taurochenodeoxycholic acid (Selleck) (5, 25, and 125 μM) for 2 h. Subsequently, the cells were stimulated with 50 ng/mL lipopolysaccharide (LPS) for 12 h. Following this, the concentration of IL-6 in the supernatant was measured by IL-6 mouse ELISA kit (Thermo Fisher) and cell viability was measured by TACS XTT cell proliferation assay kit (Trevigen, Inc.).

### Statistical analysis

Statistical analysis was performed using GraphPad Prism version 4.0 (GraphPad Software). Data are shown as mean ± SEM. Statistical significance was assessed by one-way ANOVA with Tukey and Dunnett’s posttests, or two-way ANOVA with a Bonferroni test for multiple comparisons. Kaplan-Meier survival curves were graphed and analyzed by logrank with chi-square test for multiple comparisons. *p* values < 0.05 were indicated as statistically significant.

## Results

### Antibiotic treatments reduce lethal inflammation induced by Treg deficiency

Our previous studies have shown that gut microbiota plays an important role in the development of autoimmunity in Treg-deficient SF mice [12]. We chose three antibiotics which have diverse antimicrobial spectra, looking at their impact on survival and inflammation in SF mice individually. We orally fed SF mice with ampicillin (SFA), metronidazole (SFM), or vancomycin (SFV) from 8 days of age to specified days of age (Fig. 1a). SF mice gavaged with water (as controls) uniformly died between 22 and 29 days of age (Fig. 1b). However, the lifespan of SF mice treated with antibiotics was prolonged (Fig. 1b). Remarkably, SFA mice had the longest lifespan (*p* < 0.001), whereas SFM mice had only a moderately prolonged lifespan (*p* < 0.001), compared to SFA and SFV mice (Fig. 1b). Furthermore, SFA and SFV mice had significantly reduced inflammatory infiltrates in the liver and lung (Fig. 1c, d). SFM mice had reduced inflammatory infiltrates in the liver (Fig. 1c, d). As expected, these antibiotics had no effect on the liver and lung in WT mice (Additional file 1: Figure S1). Altogether, these findings demonstrated that three antibiotics have distinct effects on the development of lethal inflammation and lifespan in Treg-deficient SF mice.

### Antibiotics remodel Treg deficiency-driven dysbiosis of the gut microbiota

**Fig. 2:**
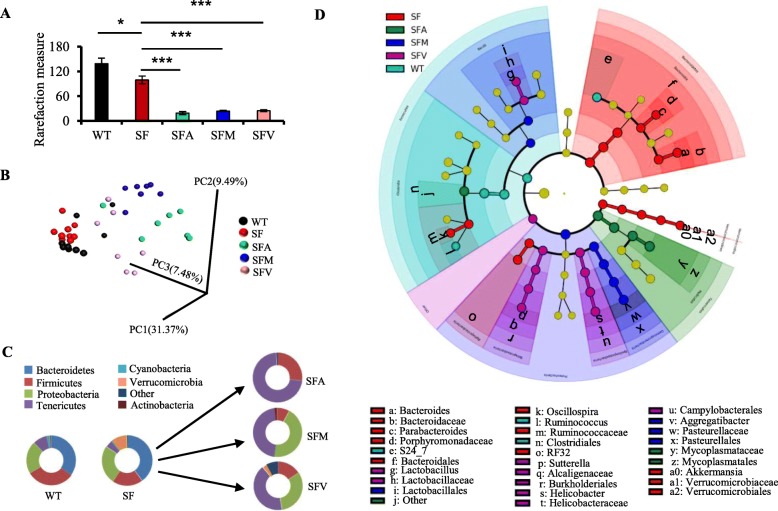
Antibiotics alter the gut microbiota in scurfy mice. **a** Gut microbial Chao1 alpha diversity analysis, comparing groups of WT, SF, SFA, SFM, and SFV mice (*n* = 6–9). **b** Unweighted UniFrac-based 3D PCoA analysis of the gut microbiota of WT, SF, SFA, SFM, and SFV mice (*n* = 6–9). c Relative abundance of the gut microbiota at the phylum level from WT, SF, SFA, SFM, and SFV mice. **d** Cladogram of the gut microbiota from WT, SF, SFA, SFM, and SFV mice. The diameter of each circle is proportional to the abundance of the taxon. The color represents which branch of the phylogenetic tree more significantly represents a certain group. Labeled nodes highlighted in each color are significantly more abundant in that branch, respectively. Nodes remaining light green indicate taxa that were not significantly differentially represented. Only differentially abundant taxa at the genus or higher taxonomic ranks were indicated. Data are presented as mean ± SEM. *p < 0.05, ***p < 0.001

To characterize the microbial populations in the feces from WT, SF, SFA, SFM, and SFV mice, we measured bacterial populations by 16S rRNA gene sequencing. Stool microbiota in SF mice was analyzed when antibiotics were orally fed by gavage from 8 to 22 days of age. The gut microbiota of SF mice was characterized by lower alpha diversity (Chao1) compared to WT mice (*p* < 0.05), consistent with our previous studies (Fig. 2a) [12]. The decreased alpha diversity (Chao1) associated with Treg deficiency was further reduced by antibiotics (*p* < 0.001) (Fig. 2a). Unweighted UniFrac-based 3D principal coordinate analysis (PCoA) also indicated a strong impact of antibiotics on the beta diversity of the gut microbiota in SF mice (Fig. 2b). Antibiotic-treated SF mice clustered distinctly from WT or SF mice (Fig. 2b). Notably, the microbial community composition of SFA mice was also distinct from that of either SFM or SFV mice (Fig. 2b). Collectively, these findings demonstrated that gut microbial dysbiosis induced by Treg deficiency could be shifted by the oral administration of antibiotics*.*

The composition of the gut microbiota of WT, SF, SFA, SFM, and SFV mice at the phylum level included seven major phyla, *Bacteroidetes*, *Firmicutes*, *Proteobacteria*, *Tenericutes*, *Cyanobacteria*, *Verrucomicrobia*, and *Actinobacteria* (Fig. 2c). The relative abundance of the phyla *Firmicutes* (*p* < 0.01) and *Tenericutes* (*p* < 0.05) was decreased, while the relative abundance of the phyla *Bacteroidetes* and *Proteobacteria* was slightly increased and the relative abundance of the phyla *Verrucomicrobia* (*p* < 0.01) was significantly increased in the stools of SF mice, compared to WT mice (Fig. 2c). Notably, ampicillin modified the effects of Treg deficiency on the relative abundance of these phyla. However, metronidazole and vancomycin modified the effects of Treg deficiency on the relative abundance of the phyla *Bacteroidetes*, *Verrucomicrobia*, and *Tenericutes* (Fig. 2c). According to the evaluation of predominant bacteria at the genus level, Treg deficiency increased the relative abundance of the genera *Bacteroides*, *Parabacteroides*, and *Akkermansia*, while antibiotics reversed the effect of Treg deficiency on these genera (Fig. 2d and Additional file 1: Figure S2). Moreover, antibiotics reversed the decreased relative abundance of the genera *Sutterella* and the family *Mycoplasmataceae* associated with Treg deficiency (Fig. 2d and Additional file 1: Figure S2). These results indicated robust differences in the membership of gut bacteria comparing WT, SF, SFA, SFM, and SFV mice.

Next, we exploited this variance in microbial composition and efficacy of antibiotics to relate features of the microbiota structure to antibiotic inhibition in SF mice. By random forests (RF) analysis, we determine the gut microbiota signatures, which result from the RF comparison of WT, SF, SFA, SFM, and SFV mice, using genus-level relative abundance data. We selected 20 significant genera as the gut microbiota signature, comparing WT, SF, SFA, SFM, and SFV mice (Additional file 1: Figure S3). Interestingly, 8 genera came from the phyla *Firmicutes* and the rest came predominantly from the phyla *Bacteroidetes* or *Proteobacteria*. The relative abundance of these phyla was altered by antibiotic treatment in SF mice (Fig. 2c). Notably, the genera *Lactobacillus* was one of the signature microbiota (Additional file 1: Figure S3), consistent with our previous studies which showed that *Lactobacillus reuteri* prolonged survival and inhibited autoimmunity in SF mice [12]. Collectively, these findings revealed unique microbiota features which likely contribute to antibiotic benefits in SF mice.

### Antibiotics alter fecal metabolome profiles in SF mice

**Fig. 3:**
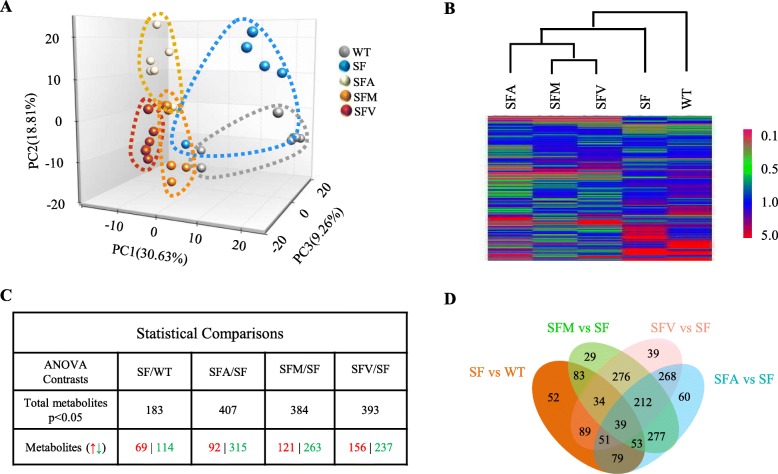
Antibiotics modulate fecal metabolomics in scurfy mice. **a** PCA clustering from fecal metabolites of WT, SF, SFA, SFM, and SFV mice (*n* = 6). **b** Hierarchical clustering from fecal metabolites of WT, SF, SFA, SFM, and SFV mice. **c** Numbers of fecal metabolites affected, either upregulated () or downregulated () (*p* < 0.05), by SF vs. WT; SFA vs. SF; SFM vs. SF or SFV vs. SF mice (*n* = 6). **d** Venn diagrams showing the overlap of fecal metabolites in **c** between different groups

Commensal bacterial metabolites can affect immune cells, which can regulate whole-body immune homeostasis [19]. To determine the effect of antibiotics on microbial metabolites in WT, SF, SFA, SFM, and SFV mice, we measured fecal metabolite profiles by non-targeted metabolomics. The PCoA and hierarchical clustering heatmap of the metabolites indicated that Treg deficiency altered fecal metabolite profiles and that antibiotic treatment had a significant impact on fecal metabolome in SF mice (SFA vs. SF, *p* < 0.001; SFM vs. SF, *p* < 0.001; SFV vs. SF, *p* < 0.001) (Fig. 3a, b). Treg deficiency significantly altered abundance of 25% (183/726) metabolites in feces compared with WT mice, while ampicillin, metronidazole, and vancomycin changed the abundance of 56% (407/726), 53% (384/726), and 54% (393/726) metabolites, respectively, compared with SF mice (Fig. 3c and Additional file 1: Table S1). Venn diagrams further demonstrated significant overlaps of the changed metabolites in SFA, SFM, and SFV mice (Fig. 3d). Among the changes, 212 metabolites were significantly affected in the three conditions SFA vs SF, SFM vs SF, and SFV vs SF (Fig. 3d). We used random forest (RF) analysis to pinpoint the group of metabolites which were most associated with antibiotics (Additional file 1: Figure S4). RF analysis of all groups resulted in an overall predictive accuracy of 93.3% (Additional file 1: Figure S5A, B). Among these altered metabolites, most of them belonged to the categories *carbohydrates* and *lipids* (Additional file 1: Figure S4). Interestingly, the metabolite with the highest mean-decrease-accuracy value was formiminoglutamate, an intermediate in histidine metabolism, which is used to identify folate/B12 deficiency that might be associated with altered microbiota (Additional file 1: Figure S5) [20]. Altogether, the above metabolomic observations revealed a profound metabolic impact of chronic antibiotics.

### Antibiotics alter cytokine expression by microbiota-associated metabolites

**Fig. 4:**
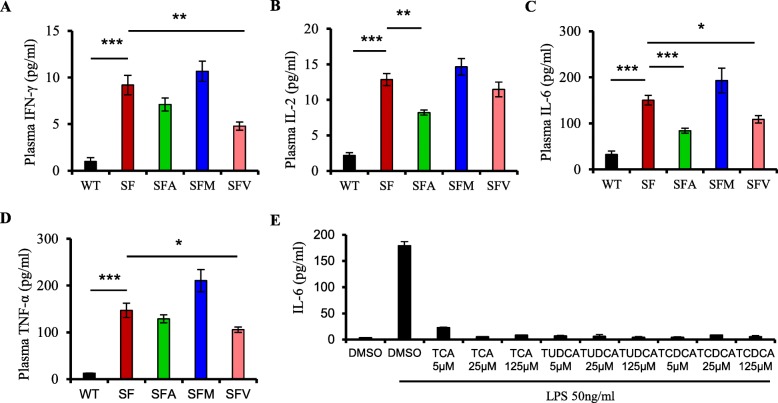
Antibiotics regulate cytokines by microbiota-associated metabolites. **a**–**d** Plasma levels of IFN-γ (**a**), IL-2(**b**), IL-6(**c**), and TNF- α(**d**) in WT, SF, SFA, SFM, and SFV mice (*n* = 6–9). **e** Level of IL-6 in RAW264.7 cells treated with DMSO (blank), LPS, taurocholic acid sodium salt hydrate (TCA), sodium tauroursodeoxycholate (TUDCA), and taurochenodeoxycholic acid (TCDCA) (*n* = 3). Data are presented as mean ± SEM. *p < 0.05, **p < 0.01, ***p < 0.001

Many cytokines are implicated in the development of lethal inflammation induced by Treg deficiency [21]. To understand which cytokines were involved in the inhibition by antibiotics of lethal inflammation in SF mice, we assessed plasma level of several cytokines. These results revealed an increased expression of cytokines, including IFN-γ, IL-2, IL-6, TNF-α, IL-4, IL-10, and IL-1β in SF mice (Fig. 4a–d and Additional file 1: Figure S6). After antibiotic treatment, vancomycin decreased plasma IFN-γ, IL-6, and TNF-α in SF mice (Fig. 4a, c, d). Ampicillin reduced plasma IL-2 and IL-6, but increased plasma IL-10 in SF mice (Fig. 4b, c and Additional file 1: Figure S6C). However, antibiotics had no impact on plasma IL-4 and IL-1β induced by Treg deficiency (Additional file 1: Figure S6A, B). Interestingly, the plasma level of IL-6 was decreased by both ampicillin and vancomycin, both of which significantly prolonged the lifespan of SF mice (Figs. 1b and 4c). Collectively, these observations suggest that antibiotics suppress lethal inflammation in SF mice in association with regulating cytokine levels, especially IL-6 expression.

To further determine the effect of the gut microbiota on plasma IL-6, we measured plasma IL-6 concentration in WT mice with ampicillin (WTA) treatment. Our results showed that ampicillin significantly reduced plasma level of IL-6 in WT mice (Additional file 1: Figure S7). We next interrogated the mechanism of antibiotic-mediated IL-6 inhibition. Bile acids, derived from host liver-gut microbiota co-metabolism, are currently receiving increased attention owing to their importance for maintaining host metabolism and immune homeostasis (Additional file 1: Figure S8A) [22]. Our results revealed that ampicillin restored fecal levels of several primary and secondary bile acids, including taurocholate (TCA), taurochenodeoxycholate (TCDCA), and tauroursodeoxycholate (TUDCA), which were reduced by Treg deficiency (Additional file 1: Figure S8B). To assess whether these bile acids contribute to the reduced IL-6 induced by antibiotics, we assessed the effect of these bile acids on IL-6 expression in RAW264.7 cells. Our studies showed that these bile acids significantly inhibited IL-6 expression (Fig. 4e). However, treatment with bile acids did not reduce the cell viability of macrophages in vitro (Additional file 1: Figure S8C). Our findings suggest that antibiotic-modulated microbiota regulates IL-6 expression at least in part by altering the bile acid pool in SF mice.

### IL-6 blockade suppresses lethal inflammation in SF mice

**Fig. 5:**
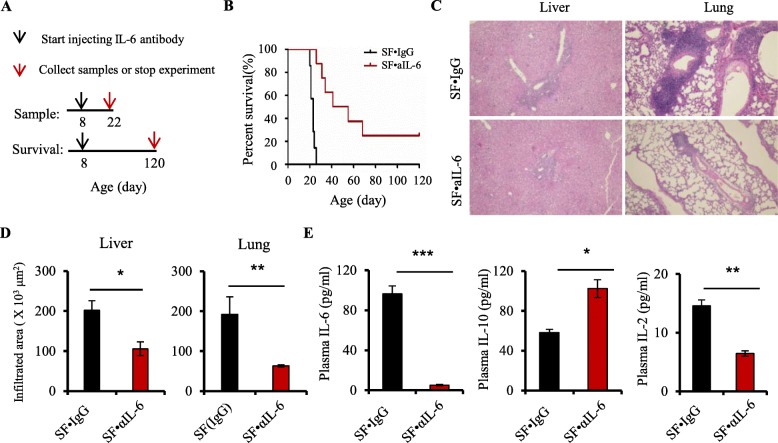
IL-6 blockade increases survival and inhibits inflammation in scurfy mice. **a** Scheme for IL-6 antibody treatment on SF mice for sample analysis and survival observation. **b** Survival curves of SF mice with IgG (SF∙IgG) or IL-6 antibody (SF∙aIL-6) treatment (*n* = 7–8). **c** Representative H&E staining of the liver and lung from SF∙IgG and SF∙aIL-6 mice (*n* = 6–9). **d** Quantitation of inflammatory infiltrates in the liver and lung of SF∙IgG and SF∙aIL-6 mice (*n* = 6–9). **e** Plasma levels of IL-6, IL-10, and IL-2 in SF∙IgG and SF∙aIL-6 mice (*n* = 6–9). Data are presented as mean ± SEM. *p < 0.05, **p < 0.01, ***p < 0.001

Given that plasma IL-6 expression was decreased by antibiotics, we addressed whether IL-6 blockade suppressed the lethal inflammation in SF mice. We treated SF mice with an IL-6 antibody which neutralizes the bioactivity of IL-6 in vivo (Fig. 5a). We determined the survival rate and quantified inflammation in the liver and lung of SF mice with IgG control (SF∙IgG) or IL-6 antibody (SF∙αIL-6) treatment. SF∙αIL-6 mice had significantly prolonged survival, compared to SF∙IgG mice (*p* < 0.001) (Fig. 5b). The inflammatory infiltrates in the liver and lung were significantly decreased by IL-6 antibody (Fig. 5c, d). Moreover, we measured plasma cytokines IL-6, IL-10, and IL-2 in SF∙IgG and SF∙αIL-6 mice and found that circulating IL-6 level in SF mice was depleted by IL-6 antibody treatment (Fig. 5e). IL-6 antibody treatment also reduced the pro-inflammatory cytokine IL-2 and increased the anti-inflammatory cytokine IL-10 in SF mice (Fig. 5e). Altogether, our data demonstrate that IL-6 blockade prolongs lifespan and reduces lethal inflammation in SF mice.

### IL-6 knockout protects against inflammation induced by Treg depletion in mice

**Fig. 6:**
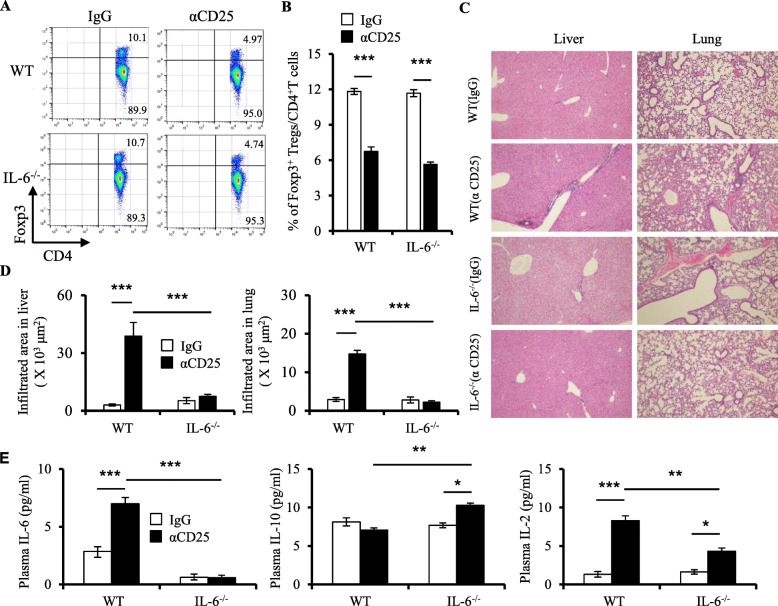
IL-6 knockout protects against inflammation induced by Treg depletion in mice. **a** Representative dot plots showing Foxp3 expression on splenic CD4+ T cells on day 14 post-injection with IgG and CD25 antibody (αCD25) in WT and IL-6^−/−^ mice (*n* = 6–8). **b** The percentage of splenic CD4+ Foxp3+ T regulatory cells on day 14 post-injection with IgG and αCD25 in WT and IL-6^−/−^ mice (*n* = 6–8). **c** Representative H&E staining of liver and lung from WT and IL-6^−/−^ mice with IgG or αCD25 treatment (*n* = 6–8). **d** Quantitation of inflammatory infiltrates in the liver and lung of WT and IL-6^−/−^ mice with IgG or αCD25 treatment (*n* = 6–8). **e** Plasma levels of IL-6, IL-10, and IL-2 in WT and IL-6^−/−^ mice with IgG or αCD25 treatment (*n* = 6–8). Data are presented as mean ± SEM. *p < 0.05, **p < 0.01, ***p < 0.001

To further explore whether IL-6 plays a critical role in the development of inflammation induced by Treg dysfunction, we used a CD25 antibody, which depletes Tregs in mice. WT and IL6^−/−^ mice were administered twice at 8 to 9 days of age with 150 mg/kg of CD25 antibody. While Foxp3 mutations result in the absence of Tregs in SF mice, CD25 antibody depleted more than 50% of Tregs in both WT and IL-6^−/−^ mice at 22 days of age (Fig. 6a, b and Additional file 1: Figure S9A-D). Interestingly, our results showed that CD25 antibody treatment induced significant inflammation in the liver and moderately inflammation in the lungs of WT mice (Fig. 6c, d). However, IL-6 knockout prevented any effects of the CD25 antibody on inflammation in the liver and lungs (Fig. 6c, d). Moreover, IL-6 knockout reversed the effect of the CD25 antibody on pro-inflammatory cytokines IL-6 and IL-2 and increased anti-inflammatory cytokine IL-10 expression in mice (Fig. 6e). These results showed that the increased IL-6 significantly contributed to the development of inflammation induced by Treg dysfunction.

## Discussion

We present in this paper evidence that daily treatment of SF mice with antibiotics is sufficient to suppress Treg deficiency-induced lethal inflammation. Antibiotic treatment prolonged lifespan, reduced inflammatory infiltrates in the liver and lungs, and decreased plasma level of pro-inflammatory cytokines which contributed to the development of lethal inflammation in SF mice. Moreover, we reveal some key mechanisms of the beneficial action of antibiotic treatment. Our findings demonstrate that changes in the gut microbiota and metabolome are linked to the benefits of antibiotic treatment in SF mice.

The IPEX syndrome and IPEX-like syndromes are due to Treg dysfunction induced by monogenic mutations [5–7]. While most of the focus in the field has been on treating this disease by stem cell transplantation [9], microbiota-based therapy of IPEX and related syndromes might be beneficial. Our studies show that gut microbiota dysbiosis contributes to the development of lethal inflammation induced by Treg deficiency. There are several findings supporting this idea. First, the composition of the gut microbiota in SF mice is different from that in WT mice (Fig. 1) [12]. Second, treatment with a single bacteria, *L. reuteri*, dramatically inhibits inflammation in SF mice [12]. Third, treatment with a single antibiotic also inhibits inflammation in SF mice (Fig. 1). Further studies will be important to investigate the downstream effects of gut microbiota dysbiosis in individuals diagnosed with IPEX syndrome or IPEX-like syndromes.

The mechanisms of the immunomodulatory effects of antibiotics are poorly understood. To reveal these mechanisms, we selected three antibiotics which have diverse antimicrobial spectrum on the intestinal microbiota. Our results showed that ampicillin and vancomycin have beneficial effects on the lethal inflammation in SF mice, but metronidazole has very moderate effects (Fig. 1). Notably, different antibiotics resulted in distinct microbiota in SF mice (Fig. 2), consistent with the previous studies [23]. The shifts in microbiota composition may only partially explain the protective effect, as these antibiotics may affect their localization within the bowel, metabolic activity, and secreted products that impact systemic immunity. We further propose a model in which antibiotics prolong lifespan and inhibit lethal inflammation by altering gut microbiota-bile acids-IL-6 axis. Consistent with this model, antibiotic treatment modulates the gut microbiota with a downstream effect of decreasing IL-6 level in SF mice (Figs. 2 and 4). In addition, antibiotic treatment reduces IL-6 expression in WT mice (Additional file 1: Figure S7). LPS-induced elevation of serum IL-6 level is also significantly reduced in germ-free mice, compared to SPF mice [24]. Elsewhere, studies have revealed that the probiotic *Lactobacillus plantarum* and a prebiotic downregulate IL-6 expression by modulating the gut microbiota [25, 26]. We suggest that the gut microbiota may be partly responsible for the plasma level of IL-6.

The gut microbiota produces numerous metabolites which can regulate host immune function and metabolism [19, 27]. The bile acids, one such class of microbial metabolites, are synthesized from cholesterol in the host liver and are further metabolized by the gut microbiota, mainly the genera *Bacteroides*, *Lactobacillus*, *Akkermansia*, *Clostridium*, *Eubacterium*, and *Escherichia*, releasing their unconjugated forms (Additional file 1: Figure S8A) [28–30]. The increased levels of genera *Bacteroides* and *Akkermansia* associated with Treg deficiency might be predicted to contribute to the reduced fecal bile acids in SF mice. Interestingly, antibiotics reduce the relative abundance of genera *Bacteroides* and *Akkermansia*, suggesting antibiotics may alter bile acid metabolism by changing the gut microbiota-liver axis. Among the most increased species of microbiota in the antibiotic-treated mouse feces were bile acid resistant or bile acid-metabolizing taxa (*Sutterella*, *Lactobacilli*, and *Enterococci*) (Additional file 1: Figures S2 and S3). Bile acids activate bile acid receptors such as FXR and TGR5, which regulate diverse immunological and metabolic pathways in the host [31, 32]. Some studies have shown that bile acid-TGR5 signaling in macrophages induces the production of IL-10, which decreases pro-inflammatory cytokines such as TNF expression, while increasing TGFβ expression and Treg populations [33, 34]. Notably, our studies reveal that antibiotics increased levels of several bile acids, including taurocholate, taurochenodeoxycholate, and tauroursodeoxycholate in SF mice (Additional file 1: Figure S9B and Additional file 1: Table S1). These bile acids significantly decreased IL-6 expression induced by LPS in cultured macrophages (Fig. 4d). Although the precise mechanism by which the gut microbiota regulates IL-6 remains to be determined, we favor the idea that bile acids may mediate communication between the gut microbiota and changes in IL-6 production in these mice.

IL-6 is pleiotropic cytokine with the ability to promote population expansion and activation of T cells, differentiation of B cells, and regulation of the acute-phase response [35, 36]. Early studies revealed that IL-6 controls the proliferation and survival of Th1/Th2 cells which play a critical role in the development of autoimmunity in both human and SF mice [36–39]. In addition, IL-6 can inhibit Treg cell function, while overexpression of IL-6 inhibits the generation of inducible Tregs but does not affect natural Tregs [40–42]. Normal physiological concentrations of IL-6 are relatively low, but these are rapidly elevated in the context of infection or autoimmunity (Fig. 4c) [43, 44]. IL-6 blockade is an effective therapy for rheumatoid arthritis in clinical practice, although some patients fail to respond to treatment [45]. Similarly, our results reveal that IL-6 blockade improves survival and lethal inflammation in SF mice (Fig. 5). In addition, IL-6 genetic deletion reverses the inflammation induced by Treg dysfunction in mice (Fig. 6). Further studies will be needed to elucidate how IL-6 contributes to the pathology in both IPEX patients and SF mice. Although there may be additional effects of antibiotic-modulated gut microbiota in suppressing inflammation in SF mice, our study suggests that the decreased IL-6 may be an important contributor to the benefits of antibiotic treatment.

## Conclusions

**Fig. 7:**
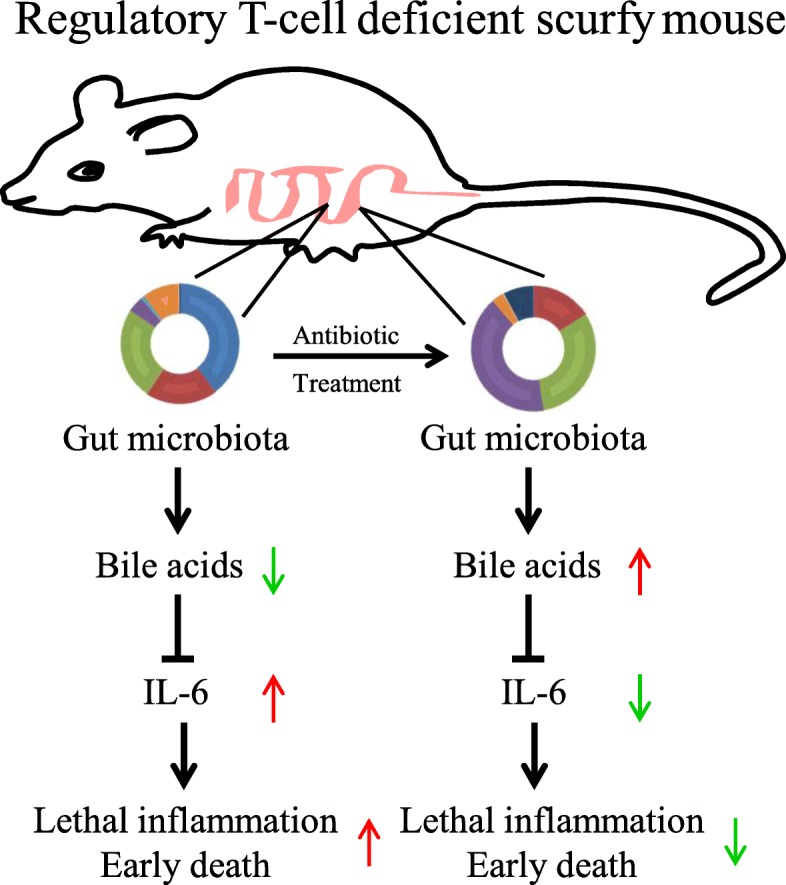
Mechanisms of protection of antibiotic-modulated microbiome against Treg deficiency-mediated lethal inflammation. Treg deficiency shapes gut microbiota and bile acid metabolism and induces IL-6 expression resulting in lethal inflammation and early death (left). Antibiotics modulate gut microbiota and bile acid metabolism and protect against Treg deficiency-induced lethal inflammation by suppressing IL-6 level (right)

Our results provide new insight into the mechanism by which a shift of the gut microbiota can negatively impact the survival and lethal inflammation in SF mice (Fig. 7). Interestingly, in our previous studies, *L. reuteri* and microbial metabolite inosine can improve the survival and lethal inflammation in SF mice [12, 46]. Here we identify different antibiotics impact the survival and lethal inflammation in SF mice despite their effect of reducing microbial diversity. In addition, the depletion of IL-6, which may be regulated by gut microbiota and bile acid metabolites, increases survival and inhibits lethal inflammation in SF mice. In summary, we propose that deeper investigation of microbiota and their products may facilitate the discovery of novel therapeutic strategies for patients with immunoregulatory diseases such as IPEX syndrome or IPEX-like syndromes.

## Supplementary information

**Additional file 1: Figure S1.** The effects of antibiotics on liver and lung in WT mice. Representative H&E staining of liver and lung from WT mice with water (WT), ampicillin (WTA), metronidazole (WTM), or vancomycin (WTV) treatment (*n* = 6–7). **Figure S2.** Relative abundance of predominant bacteria at the genus level in feces from WT, SF, SFA, SFM and SFV mice (*n* = 6–9). **Figure S3.** The microbiota signature is assessed by Random Forest Analysis. A biochemical importance plot displays the “Top 20” genera which most strongly contribute to the binning of individual samples into groups, including WT, SF, SFA, SFM and SFV mice. **Figure S4.** Random forest analysis showing a unique metabolomic signature comparing WT, SF, SFA, SFM and SFV fecal samples. A biochemical importance plot displays the “Top 40” metabolites which most strongly contribute to the binning of individual samples into groups, including WT, SF, SFA, SFM and SFV mice. **Figure S5.** The predictive accuracy of Random Forest classification. **Figure S6.** The effect of antibiotics on plasma IL-4 IL-1β and IL-10 in SF Mice. **Figure S7.** Plasma levels of IL-6 in WT mice with control (WT) or ampicillin (WTA) treatment (*n* = 6). **Figure S8**. Ampicillin alters bile acid metabolism in SF mice. **Figure S9.** The percentage of Treg cells in WT with anti-CD25 treatment, IL-6^−/−^ with anti-CD25 treatment, WT and SF mice. **Table S1.** Identification of 726 fecal metabolites and their relative quantification in WT, SF and SF mice with ampicillin(SFA), metronidazole(SFM) orvancomycin(SFV) treatment. Fold changes were calculated from 6 experimental samples from each group and subjected to ANOVA analysis.

## Data Availability

16S rRNA gene sequences of gut microbiota have been deposited in the NCBI Sequence Read Archive under BioProject accession number PRJNA548430.

## Abbreviations

CTLA-4: Cytotoxic T lymphocyte-associated antigen 4
DOCK8: Dedicator of cytokinesis protein 8
FOXP3: Forkhead box P3
IPEX: Immune dysregulation polyendocrinopathy enteropathy X-linked
ITCH: Itchy E3 ubiquitin protein ligase
LRBA: LPS-responsive and beige-like anchor protein
STAT: Signal transducer and activator of transcription
TCA: Taurocholate
TCDCA: Taurochenodeoxycholate
Treg: Regulatory T
TUDCA: Tauroursodeoxycholate
