# Supplementary material for: Antibiotic-modulated microbiome suppresses lethal inflammation and prolongs lifespan in Treg-deficient mice
Source: Microbiome. 2019 Nov 7;7:145. doi: 10.1186/s40168-019-0751-1 (PMC6839243; doi:10.1186/s40168-019-0751-1)
Supplement: Supplementary file 1 — Additional file 1: Figure S1. The effects of antibiotics on liver and lung in WT mice. Representative H&E staining of liver and lung from WT mice with water (WT), ampicillin (WTA), metronidazole (WTM), or vancomycin (WTV) treatment (n = 6–7). Figure S2. Relative abundance of predominant bacteria at the genus level in feces from WT, SF, SFA, SFM and SFV mice (n = 6–9). Figure S3. The microbiota signature is assessed by Random Forest Analysis. A biochemical importance plot displays the “Top 20” genera which most strongly contribute to the binning of individual samples into groups, including WT, SF, SFA, SFM and SFV mice. Figure S4. Random forest analysis showing a unique metabolomic signature comparing WT, SF, SFA, SFM and SFV fecal samples. A biochemical importance plot displays the “Top 40” metabolites which most strongly contribute to the binning of individual samples into groups, including WT, SF, SFA, SFM and SFV mice. Figure S5. The predictive accuracy of Random Forest classification. Figure S6. The effect of antibiotics on plasma IL-4 IL-1β and IL-10 in SF Mice. Figure S7. Plasma levels of IL-6 in WT mice with control (WT) or ampicillin (WTA) treatment (n = 6). Figure S8. Ampicillin alters bile acid metabolism in SF mice. Figure S9. The percentage of Treg cells in WT with anti-CD25 treatment, IL-6−/− with anti-CD25 treatment, WT and SF mice. Table S1. Identification of 726 fecal metabolites and their relative quantification in WT, SF and SF mice with ampicillin(SFA), metronidazole(SFM) orvancomycin(SFV) treatment. Fold changes were calculated from 6 experimental samples from each group and subjected to ANOVA analysis. [file 40168_2019_751_MOESM1_ESM.pdf]

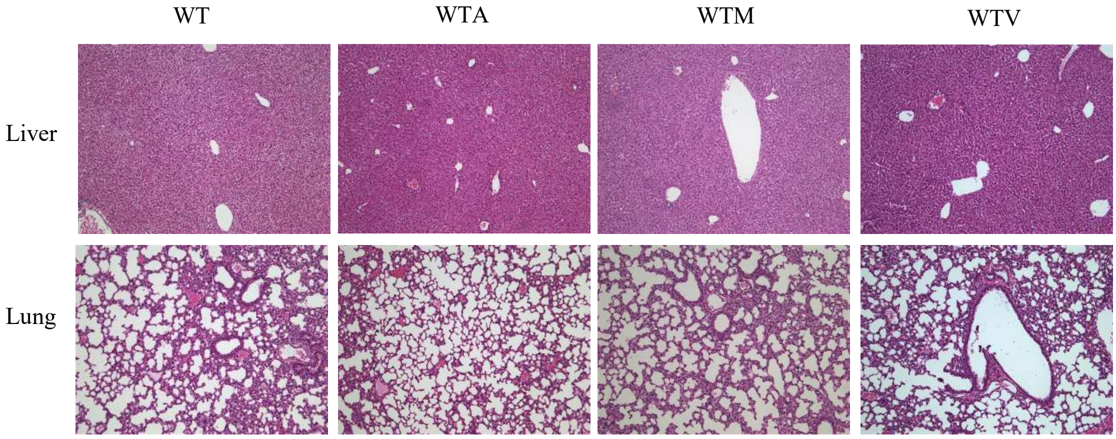

Fig S1. The effects of antibiotics on liver and lung in WT mice. Representative H&E staining of liver and lung from WT mice with water (WT), ampicillin (WTA), metronidazole (WTM), or vancomycin (WTV) treatment (n=6-7).

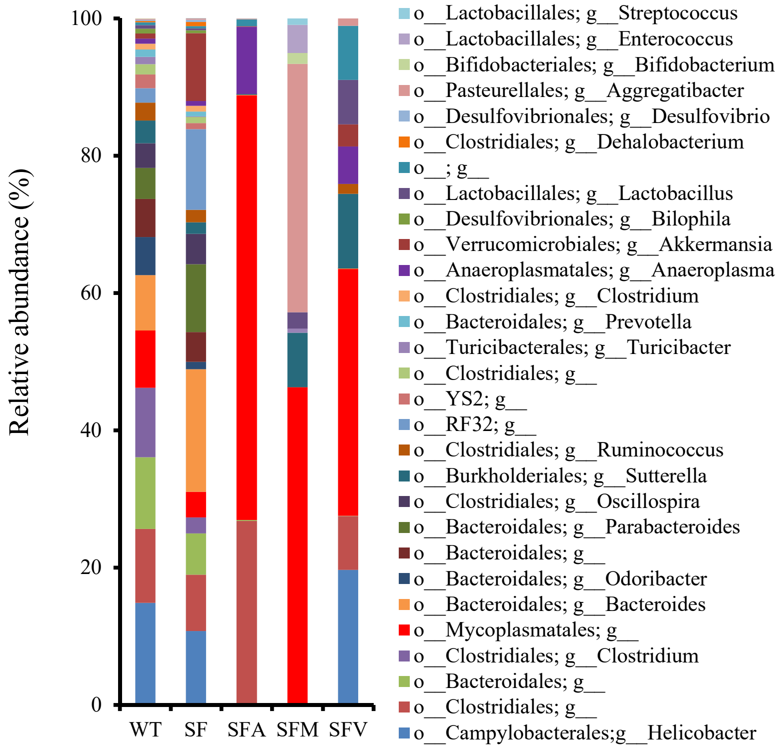

Fig S2. Relative abundance of predominant bacteria at the genus level in feces from WT, SF, SFA, SFM and SFV mice (n=6-9).

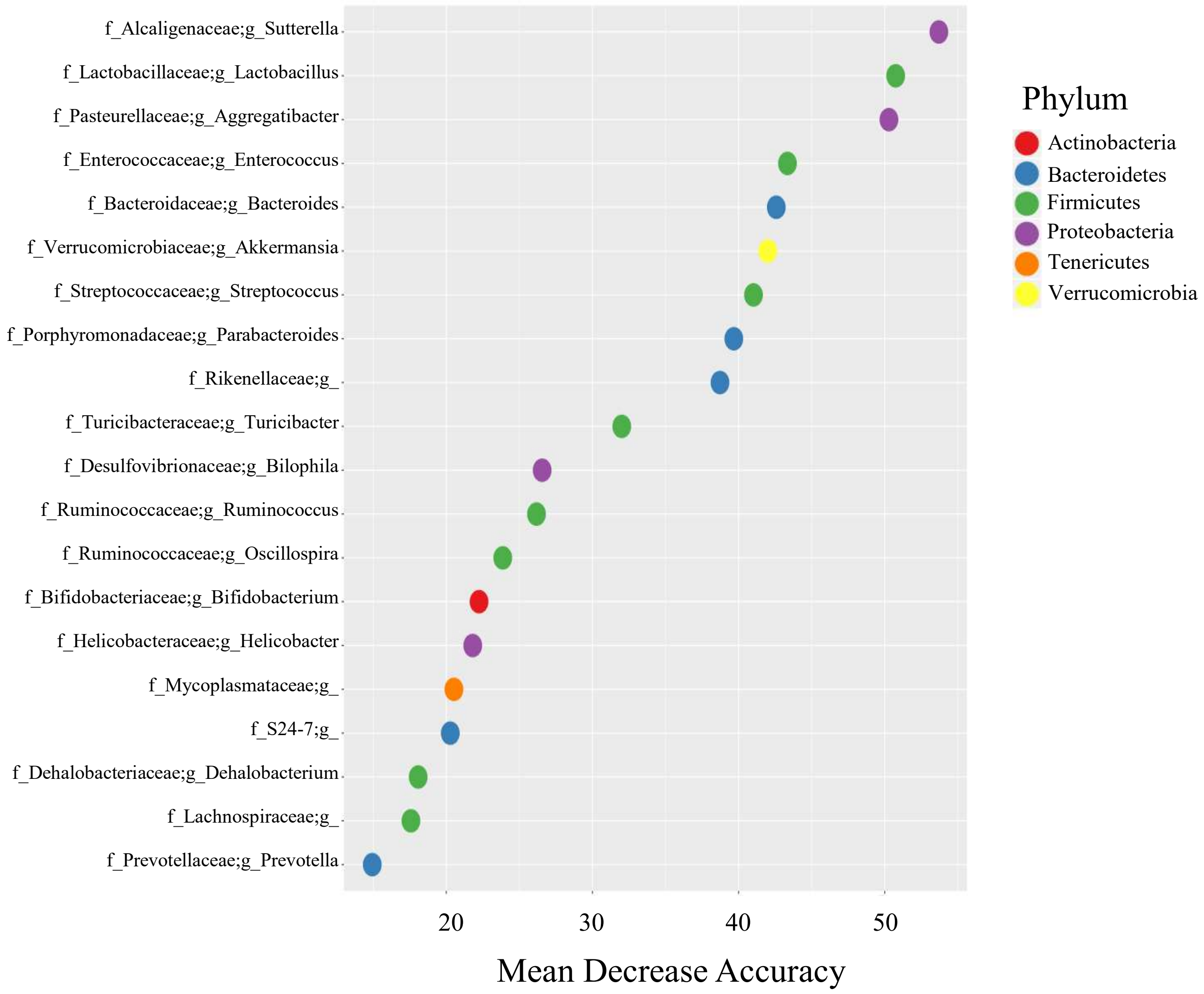

Fig S3. The microbiota signature is assessed by Random Forest Analysis. A biochemical importance plot displays the "Top 20" genera which most strongly contribute to the binning of individual samples into groups, including WT, SF, SFA, SFM and SFV mice.

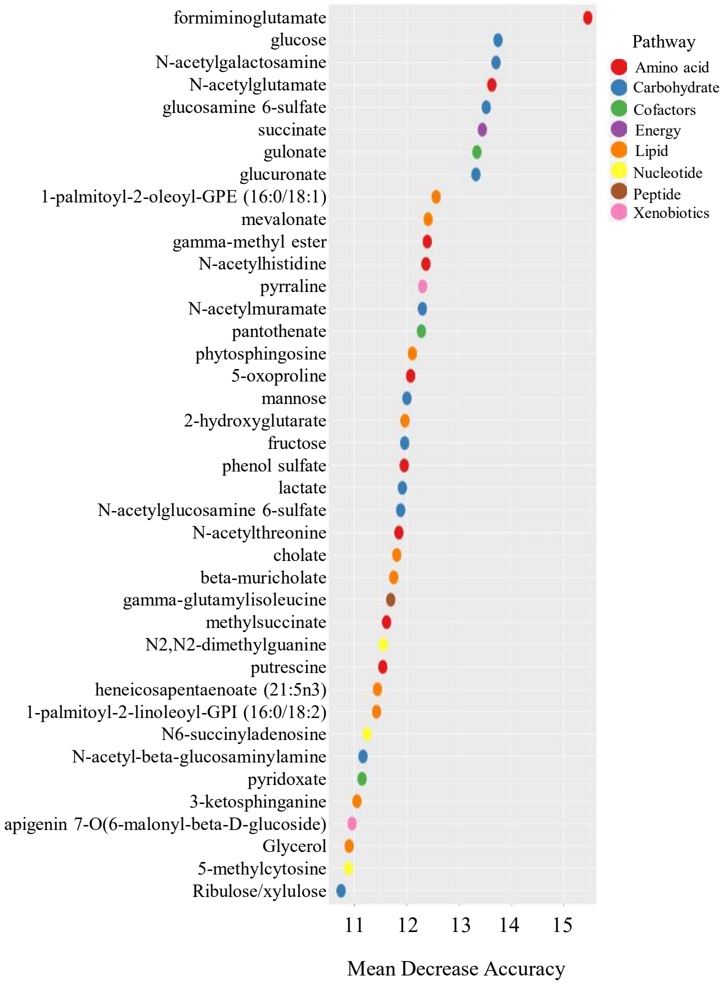

Fig S4. Random forest analysis showing a unique metabolomic signature comparing WT, SF, SFA, SFM and SFV fecal samples. A biochemical importance plot displays the “Top 40” metabolites which most strongly contribute to the binning of individual samples into groups, including WT, SF, SFA, SFM and SFV mice.

A

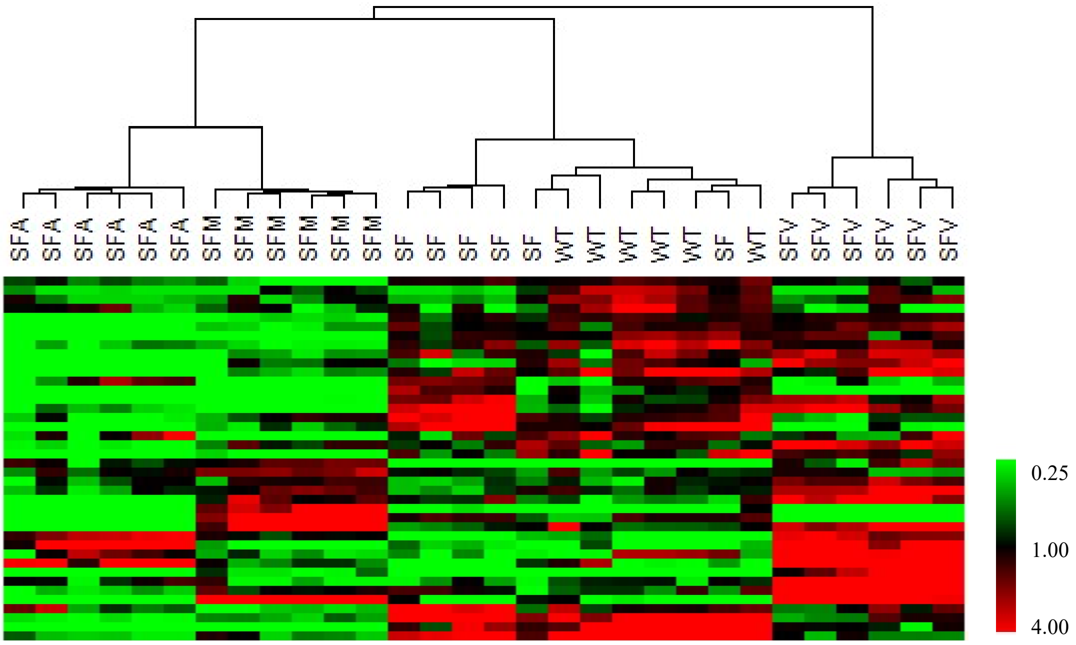

B

Predicted Group

Actual Group

	WT	SF	SFA	SFM	SFV	Class Error
WT	6	2	0	0	0	0.000
SF	0	4	0	0	0	0.333
SFA	0		6	0	0	0.000
SFM	0		0	6	0	0.000
SFV	0		0	0	6	0.000
**Predictive accuracy = 93.3%**						

Fig S5. The predictive accuracy of Random Forest classification. (A) Hierarchical clustering of WT, SF, SFA, SFM and SFV mice with the top 40 metabolites in online supplementary figure 4. Each lane represents a different mouse. The scale bar indicates the relative abundance of metabolites (n=6). (B) Random Forest classification using the top 40 of metabolites in (A) gave a predictive accuracy of 93.3%.

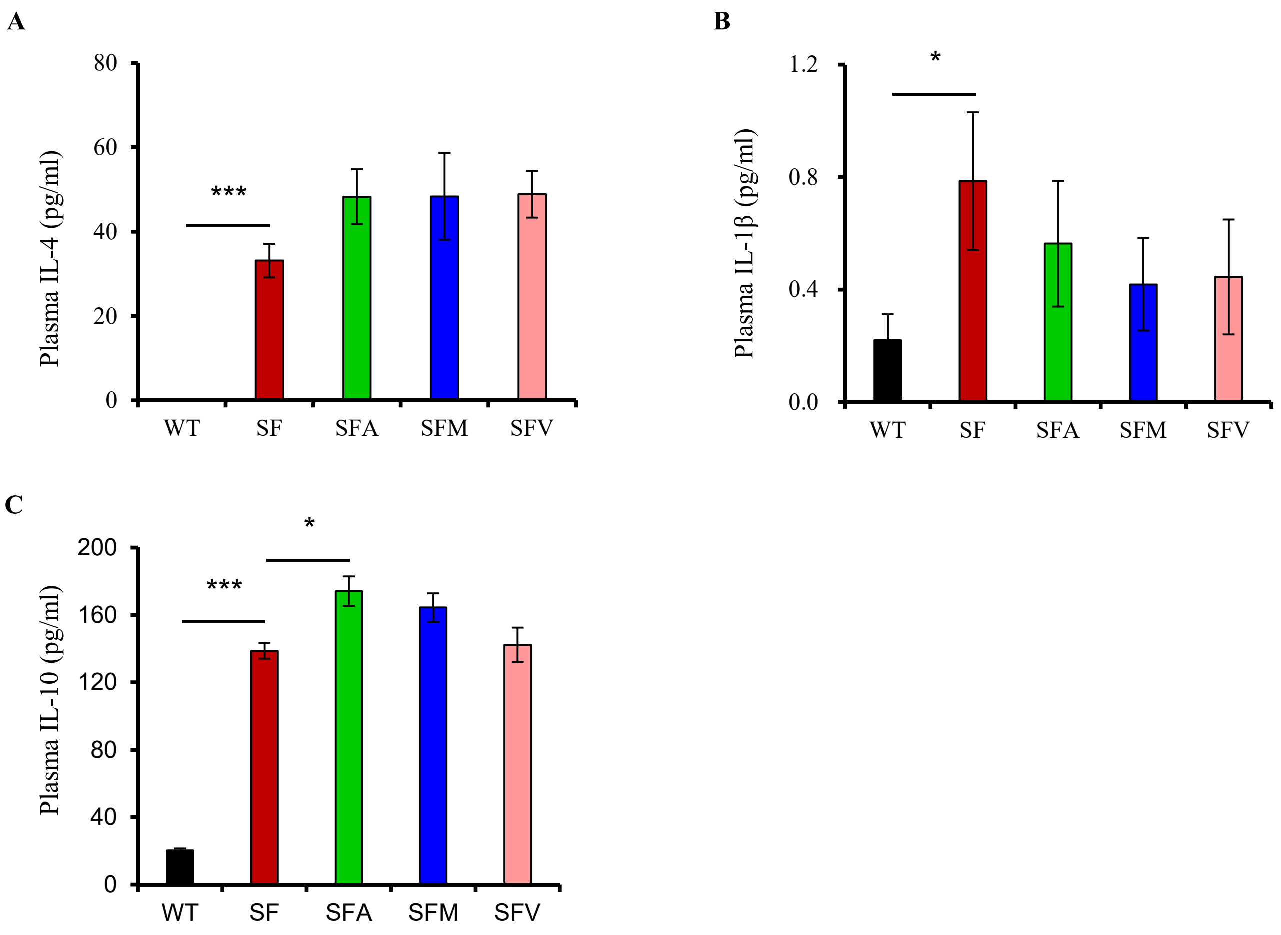

Fig S6. The effect of antibiotics on plasma IL-4 IL-1β and IL-10 in SF Mice. Plasma levels of IL-4 (A), IL-1β (B) and IL-10 (C) in WT, SF, SFA, SFM and SFV mice (n=6-7). Data are presented as mean ± SEM. * $p < 0.05$, *** $p < 0.001$.

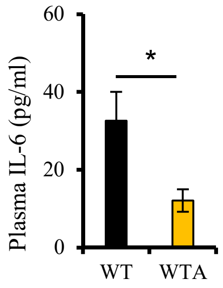

Fig S7. Plasma levels of IL-6 in WT mice with control (WT) or ampicillin (WTA) treatment (n=6). Data are presented as mean ± SEM. * $p < 0.05$.

A

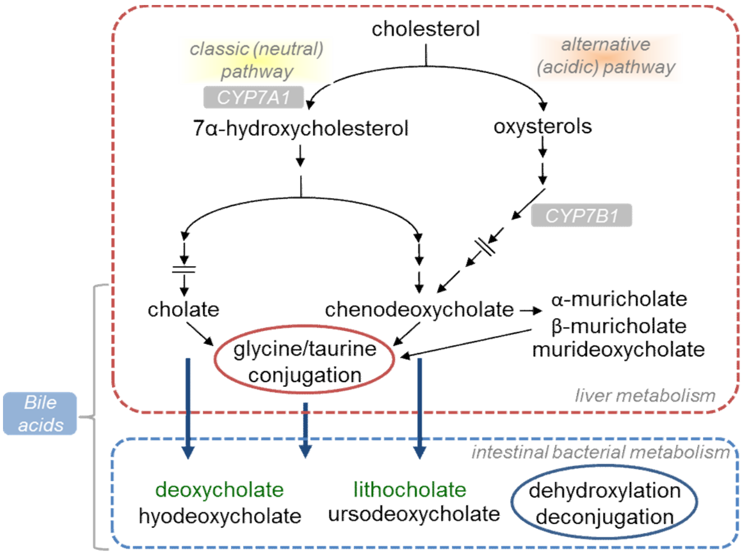

C

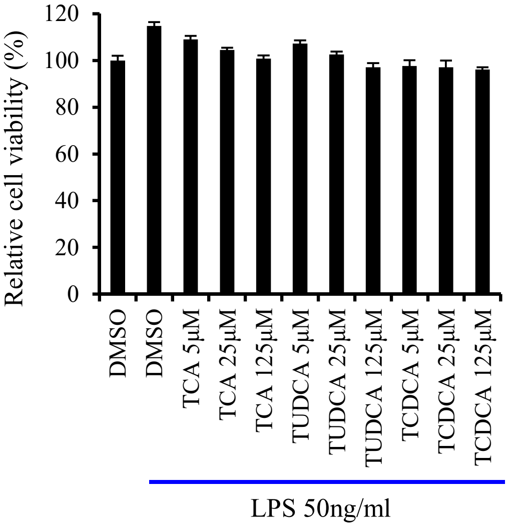

B

Sub Pathway	Biochemical Names	SF / WT	SFA / SF
Primary Bile Acid Metabolism	cholate	0.31	0.00
	glycocholate	0.19	0.89
	taurocholate	0.00	26.74
	chenodeoxycholate	0.12	0.04
	taurochenodeoxycholate	0.01	16.79
	beta-muricholate	0.39	0.00
	alpha-muricholate	0.20	0.00
	tauro-beta-muricholate	0.00	17.61
	cholate sulfate	0.58	0.18
Secondary Bile Acid Metabolism	deoxycholate	0.60	0.00
	6-beta-hydroxylithocholate	0.49	0.03
	lithocholate	0.88	0.03
	ursodeoxycholate	0.22	0.17
	tauroursodeoxycholate	0.00	10.20
	dehydrolithocholate	1.09	0.04
	7,12-diketolithocholate	1.30	0.16
	6-oxolithocholate	0.35	0.91
	hyocholate	1.64	0.02
	3-dehydrocholate	0.63	0.02
	12-dehydrocholate	2.83	0.01
	taurocholenate sulfate	0.66	22.80
	7-ketodeoxycholate	1.04	0.00
	3b-hydroxy-5-cholenoic acid	1.59	0.05
	taurochenodeoxycholate sulfate	0.30	4.32
	ursocholate	0.53	0.09

Green: indicates significance $p \leq 0.05$, metabolite ratio of < 1.00
Light Green: indicates significance 0.05<p<0.10, metabolite ratio of < 1.00
Red: indicates significance $p \leq 0.05$, metabolite ratio of ≥ 1.00
Light Red: indicates significance 0.05<p<0.10, metabolite ratio of ≥ 1.00
Non-colored text and cell: indicates significance p≥0.10,

Fig S8. Ampicillin alters bile acid metabolism in SF mice. (A) Scheme of bile acid metabolism pathway. (B) Heat map showing fold changes of fecal bile acid metabolites in SF *vs.* WT and SFA *vs.* SF (n=6). (C) The cell viability of macrophages treated with taurocholate (TCA), taurochenodeoxycholate (TCDCA), or tauroursodeoxycholate (TUDCA) at the indicated concentration (n=4).

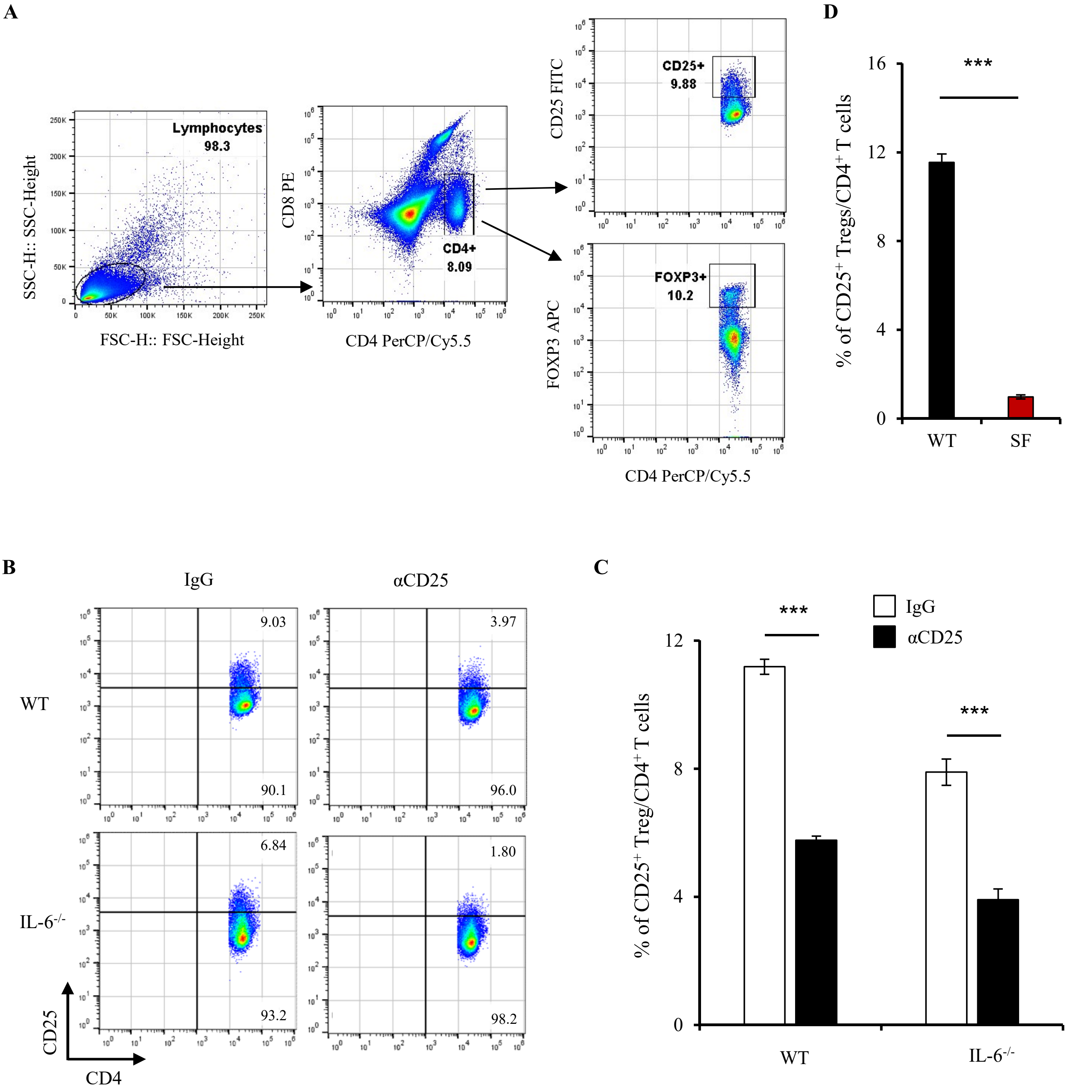

Fig S9. The percentage of Treg cells in WT with anti-CD25 treatment, IL-6$^{-/-}$ with anti-CD25 treatment, WT and SF mice. (A) The definition of CD4$^+$ Foxp3$^+$ Treg cells and CD4$^+$ CD25$^+$ Treg cells in WT lymphocyte populations are shown by representative flow cytometry plots. (B) Representative dot plots showing CD25 expression on splenic CD4$^+$ T cells on day 14 post-injection with IgG or CD25 antibody (αCD25) in in WT and IL-6$^{-/-}$ mice. (C) The percentage of splenic CD4$^+$ CD25$^+$ Treg cells on day 14 post-injection with IgG and α CD25 in WT and IL-6$^{-/-}$ mice (n=6-8). (D) The percentage of splenic CD4$^+$ Foxp3$^+$ Treg cells in WT and SF mice at 22 days of age (n=6-7). Data are presented as mean ± SEM. *** $p< 0.001$.

Table S1. Identification of 726 fecal metabolites and their relative quantification in WT, SF and SF mice with ampicillin (SFA), metronidazole (SFM) or vancomycin (SFV) treatment. Fold changes were calculated from 6 experimental samples from each group and subjected to ANOVA analysis.

**Biochemical name**	**SF *vs* WT**	**SFA *vs* SF**	**SFV *vs* SF**	**SFM *vs* SF**
glycine	0.93	**0.62**	1.27	0.84
N-acetylglycine	0.65	**0.10**	**2.04**	**0.54**
sarcosine	1.08	**0.03**	**0.03**	**0.03**
dimethylglycine	**3.97**	**0.31**	**0.36**	**0.36**
betaine	1.30	1.14	1.10	**1.58**
betaine aldehyde	**1.20**	0.64	**0.18**	1.20
serine	0.95	0.81	**1.38**	**0.77**
N-acetylserine	0.73	**0.38**	1.76	0.78
threonine	1.06	0.75	**1.54**	**0.60**
N-acetylthreonine	0.78	1.19	**3.72**	**2.09**
alanine	0.93	**0.39**	0.84	**0.50**
N-acetylalanine	0.86	**1.66**	**2.96**	**3.67**
aspartate	1.10	**0.47**	**0.69**	**0.50**
N-acetylaspartate (NAA)	**5.08**	**0.01**	**0.03**	0.49
asparagine	0.66	**5.39**	3.05	1.97
N-acetylasparagine	1.03	**0.16**	0.60	**0.50**
glutamate	1.16	**0.29**	**0.72**	**0.50**
glutamine	0.81	0.80	**1.79**	0.86
N-acetylglutamate	1.51	**0.01**	**0.08**	**0.14**
N-acetylglutamine	1.25	**0.11**	**0.60**	**0.19**
glutamate, gamma-methyl ester	0.87	1.22	1.03	**3.11**
N-acetyl-aspartyl-glutamate (NAAG)	**0.63**	**1.68**	**2.55**	**2.68**
carboxyethyl-GABA	**0.66**	**0.38**	0.79	0.68
S-1-pyrroline-5-carboxylate	0.94	0.73	0.73	1.10
histidine	0.84	0.83	**1.89**	**0.44**
N-acetylhistidine	0.72	0.88	**7.06**	**2.35**
N-acetyl-3-methylhistidine*	**0.50**	1.63	**2.56**	**2.17**
N-acetyl-1-methylhistidine*	0.68	1.38	**3.25**	**2.31**
hydantoin-5-propionic acid	**2.16**	**0.12**	**0.12**	**0.12**
trans-urocanate	0.59	0.64	**0.05**	**0.18**
imidazole propionate	0.86	**0.01**	**1.33**	**2.04**
formiminoglutamate	1.17	**0.04**	**0.04**	**3.28**
imidazole lactate	3.00	**0.05**	**1.41**	0.65
histamine	**0.22**	**0.24**	**2.06**	1.71
1-methylhistamine	1.81	1.08	1.10	0.66
1-methylimidazoleacetate	1.72	0.70	0.76	0.79
4-imidazoleacetate	1.69	**0.46**	**0.47**	**0.27**
N-acetylhistamine	0.51	**0.02**	**0.27**	0.53
lysine	**0.75**	**0.68**	**1.28**	0.98
N2-acetyllysine	2.12	**0.20**	**0.25**	0.55
N6-acetyllysine	0.90	**0.18**	1.51	0.77
N2,N6-diacetyllysine	0.93	0.91	**2.67**	**1.99**

N6,N6,N6-trimethyllysine	0.66	0.81	1.67	1.21
5-hydroxylysine	0.50	0.97	1.20	2.24
saccharopine	1.58	0.32	0.81	1.12
glutarylcarnitine (C5-DC)	0.88	2.61	3.86	3.25
pipecolate	0.97	0.02	0.49	0.13
6-oxopiperidine-2-carboxylate	0.71	0.39	0.39	0.62
cadaverine	6.30	0.05	1.73	0.15
N-acetyl-cadaverine	1.48	0.01	2.27	1.05
5-aminovalerate	0.97	0.01	0.01	0.01
N-trimethyl 5-aminovalerate	1.55	0.03	0.03	0.04
phenylalanine	0.73	0.66	1.45	0.81
N-acetylphenylalanine	0.71	0.36	4.10	1.67
phenylpyruvate	0.92	0.05	0.41	0.30
phenyllactate (PLA)	0.50	0.04	10.07	3.79
phenethylamine	1.06	1.25	3.82	1.02
phenylacetate	1.28	0.04	0.06	0.04
4-hydroxyphenylacetate	0.52	0.05	0.09	0.05
tyrosine	0.84	0.49	1.20	0.60
N-acetyltyrosine	0.93	0.16	2.75	1.11
tyramine	0.55	0.40	0.51	1.41
4-hydroxyphenylpyruvate	0.88	0.49	0.38	0.20
3-(4-hydroxyphenyl)lactate	0.45	0.88	11.71	3.39
phenol sulfate	0.52	0.43	0.43	28.50
O-methyltyrosine	0.90	0.26	0.55	0.63
gentisate	0.55	0.02	0.09	0.20
N-formylphenylalanine	0.85	0.35	0.65	0.33
4-hydroxycinnamate sulfate	0.40	1.49	5.28	3.02
tryptophan	0.76	0.94	1.51	1.07
N-acetyltryptophan	0.96	0.39	2.11	2.29
C-glycosyltryptophan	1.71	2.94	4.38	3.22
kynurenine	1.22	0.87	0.64	1.11
N-acetylkynurenine (2)	3.66	0.40	0.91	0.64
kynurenate	1.79	0.93	0.70	1.40
N-formylanthranilic acid	1.12	0.18	0.31	0.26
xanthurenate	0.75	0.57	1.04	1.19
serotonin	0.59	0.34	0.46	0.33
5-hydroxyindoleacetate	1.12	0.37	0.30	0.45
indolelactate	0.22	0.09	6.17	4.80
indoleacetate	0.54	0.16	0.11	0.13
indolepropionate	0.45	0.22	0.22	0.22
indoleacetylglycine	1.00	4.50	2.27	2.14
3-indoxyl sulfate	1.23	1.34	0.39	1.29
leucine	0.79	0.76	1.50	0.97
N-acetylleucine	1.05	0.48	1.47	1.66
4-methyl-2-oxopentanoate	0.89	0.01	0.16	0.10
alpha-hydroxyisocaproate	0.51	0.03	5.12	4.42
isovalerate	0.87	0.18	0.08	0.14
isovalerylglycine	0.79	0.47	0.80	0.88
isovalerylcarnitine (C5)	1.46	2.01	3.53	4.65
beta-hydroxyisovalerate	0.67	0.45	0.37	0.57

isoleucine	0.92	0.71	1.48	0.86
N-acetylisoleucine	1.13	0.46	1.79	1.60
3-methyl-2-oxovalerate	1.01	0.01	0.16	0.09
alpha-hydroxyisovalerate	0.53	0.05	8.04	5.48
2-methylbutyrylcarnitine (C5)	3.45	6.13	9.33	10.12
2-methylbutyrylglycine	0.68	0.83	1.26	1.37
ethylmalonate	0.70	0.31	0.27	0.60
methylsuccinate	0.69	0.05	0.08	0.08
valine	0.88	0.69	1.40	0.84
N-acetylvaline	0.80	1.73	2.66	2.74
3-methyl-2-oxobutyrate	0.84	0.03	0.12	0.07
2-hydroxy-3-methylvalerate	0.59	0.02	7.56	5.01
isobutyrylcarnitine (C4)	4.41	2.49	0.32	4.77
isobutyrylglycine	0.55	0.31	0.58	0.43
3-hydroxyisobutyrate	1.27	0.57	0.61	1.21
valine betaine	0.22	0.88	1.98	1.95
methionine	0.79	0.52	0.98	0.63
N-acetylmethionine	0.60	1.08	4.29	1.01
N-formylmethionine	1.02	0.12	0.40	0.40
methionine sulfoxide	0.74	0.27	0.74	0.37
N-acetylmethionine sulfoxide	0.59	1.76	1.96	1.36
4-methylthio-2-oxobutanoate	0.85	0.15	0.28	0.25
alpha-ketobutyrate	0.67	0.48	1.01	0.48
cysteine	0.61	1.39	4.40	2.09
N-acetylcysteine	6.14	0.18	0.30	0.24
S-methylcysteine	0.99	0.24	0.43	0.61
cysteine s-sulfate	0.30	4.08	4.45	1.70
cystine	1.30	5.89	5.00	2.59
cysteine sulfinic acid	0.77	0.92	2.42	1.63
hypotaurine	1.23	0.29	0.42	0.46
taurine	0.92	0.27	0.37	0.80
N-acetyltaurine	0.22	6.30	10.51	12.26
3-sulfo-L-alanine	0.67	0.17	0.27	0.17
arginine	0.68	0.88	1.18	1.54
argininosuccinate	1.10	0.93	0.62	0.83
urea	1.32	5.25	0.86	3.11
ornithine	1.08	1.45	0.72	1.66
2-oxoarginine*	0.78	2.20	0.74	0.39
citrulline	0.57	0.28	1.08	0.31
homocitrulline	2.10	0.08	0.18	0.12
proline	0.80	0.76	2.79	0.89
dimethylarginine (SDMA + ADMA)	0.85	0.60	1.37	0.99
N-acetylarginine	0.68	0.59	2.99	1.10
N-acetylcitrulline	1.96	0.11	0.49	0.25
N-acetylproline	0.62	0.54	1.04	1.23
N-delta-acetylornithine	2.13	0.02	0.16	0.16
N-alpha-acetylornithine	1.78	0.02	0.06	0.38
trans-4-hydroxyproline	0.30	0.58	3.00	2.25
pro-hydroxy-pro	0.46	2.40	0.36	3.73
N-methylproline	0.94	0.01	0.01	0.02

N-monomethylarginine	0.53	0.58	1.06	1.69
argininate*	0.62	0.24	**3.47**	**0.14**
guanidinoacetate	1.09	0.64	0.72	1.01
creatine	1.49	1.26	**1.70**	**2.13**
creatinine	**0.28**	**1.82**	**4.04**	**4.53**
agmatine	**0.68**	0.25	0.43	0.32
putrescine	1.23	0.30	**26.92**	**29.07**
spermidine	2.69	1.56	0.83	1.38
N1,N12-diacetylspermine	3.83	0.55	**4.74**	**1.88**
5-methylthioadenosine (MTA)	1.34	**5.62**	0.49	0.89
N-acetylputrescine	3.56	**0.02**	**3.53**	**1.79**
4-acetamidobutanoate	2.74	**0.08**	0.48	0.41
(N(1) + N(8))-acetylspermidine	3.05	**2.57**	**3.25**	**2.24**
1-methylguanidine	**0.50**	**0.39**	0.73	0.73
4-guanidinobutanoate	**0.39**	1.51	2.56	**2.35**
cysteinylglycine	0.75	0.72	1.30	**1.59**
5-oxoproline	0.53	**0.23**	**7.98**	**7.24**
2-hydroxybutyrate/2-hydroxyisobutyrate	0.65	**0.42**	**1.54**	**1.94**
gamma-glutamylalanine	0.77	**0.10**	0.56	**0.15**
gamma-glutamylglutamate	1.30	**0.05**	**0.09**	0.32
gamma-glutamylglutamine	0.99	**0.29**	**0.42**	0.74
gamma-glutamylglycine	0.54	0.40	0.72	0.58
gamma-glutamylhistidine	0.69	**0.24**	0.75	0.58
gamma-glutamylisoleucine*	1.39	**0.08**	**4.69**	**1.80**
gamma-glutamylleucine	**0.55**	**0.42**	**2.05**	1.04
gamma-glutamyl-alpha-lysine	0.63	**0.25**	0.56	0.57
gamma-glutamyl-epsilon-lysine	0.61	**6.09**	**6.81**	**3.51**
gamma-glutamylmethionine	0.86	**0.25**	**0.29**	1.22
gamma-glutamylphenylalanine	0.63	**0.31**	**0.35**	0.78
gamma-glutamylthreonine	0.89	**0.15**	**0.33**	**0.23**
gamma-glutamyltyrosine	0.77	**0.23**	**0.27**	0.68
gamma-glutamylvaline	0.67	**0.14**	1.22	1.15
alanylleucine	0.33	0.39	0.68	0.40
glutaminylleucine	**0.19**	0.43	0.76	0.43
glycylisoleucine	0.78	**0.27**	1.57	**0.35**
glycylleucine	0.77	**0.24**	0.71	**0.30**
glycylvaline	0.66	**0.41**	1.31	**0.47**
histidylalanine	**0.41**	0.80	**4.40**	0.88
isoleucylglycine	0.55	**0.16**	0.58	**0.12**
leucylalanine	0.32	1.07	**1.84**	1.18
leucylglycine	0.46	**0.37**	1.09	0.75
lysylleucine	**0.37**	**0.29**	0.83	**0.10**
phenylalanylalanine	0.36	**3.41**	**2.95**	**4.52**
phenylalanylglycine	**0.28**	0.96	**2.11**	0.42
prolylglycine	0.40	**1.98**	0.55	**3.00**
threonylphenylalanine	**0.29**	0.14	0.27	0.15
tryptophylglycine	**0.13**	1.83	2.05	2.07
tyrosylglycine	**0.37**	**2.67**	**3.38**	**2.33**
valylglutamine	**0.41**	0.48	**2.16**	0.24
valylglycine	**0.38**	**0.12**	0.94	0.62

valylleucine	0.34	0.36	0.63	0.42
leucylglutamine*	0.24	0.80	4.79	0.63
1,5-anhydroglucitol (1,5-AG)	0.93	0.53	1.20	0.87
glucose	0.77	0.09	1.03	0.01
pyruvate	0.89	0.27	1.94	1.56
lactate	1.06	0.19	2.96	0.96
glycerate	0.88	0.10	0.58	0.38
sedoheptulose-7-phosphate	0.23	0.02	0.02	0.02
ribose	1.65	0.21	0.35	0.05
ribitol	0.51	0.57	1.76	1.71
ribonate	0.67	4.02	7.28	6.41
xylose	0.47	0.02	0.18	0.05
arabinose	0.41	0.16	0.20	0.13
fucose	1.48	0.26	6.96	0.35
arabitol/xylitol	0.28	2.34	3.62	4.22
ribulose/xylulose	0.68	0.22	0.92	0.17
arabonate/xylonate	0.38	3.20	6.02	6.85
sedoheptulose	0.90	1.02	2.46	2.25
maltose	0.09	0.02	0.25	0.04
stachyose	0.50	14.63	0.26	0.21
lactobionate	0.35	7.62	9.07	0.63
3-sialyllactose	1.00	53.00	6.66	2.78
6'-sialyllactose	0.39	526.89	105.78	276.41
3-fucosyllactose	0.51	14.36	1.58	13.99
sucrose	0.54	0.55	0.81	0.09
raffinose	0.41	13.90	0.07	0.06
fructose	0.64	1.61	1.07	0.06
mannitol/sorbitol	0.53	8.85	9.20	10.79
mannose	1.73	0.78	0.22	0.01
galactitol (dulcitol)	0.44	3.27	4.40	8.25
galactonate	0.53	11.18	16.61	19.21
glucosamine 6-sulfate	1.00	1.00	146.03	4.10
glucuronate	0.56	16.73	17.15	5.60
diacetylchitobiose	1.00	4.86	4.89	1.60
N-acetylglucosamine 6-sulfate	0.93	0.35	22.12	1.16
N-acetylneuraminate	1.13	2.23	15.33	0.53
N-acetyl-beta-glucosaminylamine	4.53	0.18	0.17	0.04
N-acetylmuramate	2.06	0.04	1.03	0.09
3'-a-sialyl-N-acetyllactosamine	1.00	4.63	1.00	1.00
3'-beta-Sialyl-N-acetyllactosamine	1.00	2.88	1.00	1.84
6-sialyl-N-acetyllactosamine	1.00	3.18	1.84	1.59
N-acetylglucosaminylasparagine	0.91	0.16	1.60	1.34
erythronate*	0.52	0.71	2.91	2.53
N-acetylglucosamine/N-acetylgalactosamine	2.72	0.12	1.16	0.00
N6-carboxymethyllysine	0.40	1.13	2.42	3.06
citrate	0.48	0.54	0.53	0.69
aconitate [cis or trans]	0.43	2.29	3.38	4.04
alpha-ketoglutarate	1.04	0.02	0.11	0.07
succinylcarnitine (C4-DC)	0.93	2.16	4.12	3.08
succinate	0.72	0.02	1.27	0.28

fumarate	1.20	0.01	1.29	1.10
malate	1.32	0.02	0.53	0.39
tricarballylate	0.40	0.18	0.53	0.19
2-methylcitrate/homocitrate	0.65	0.05	0.15	0.09
phosphate	1.23	0.08	0.36	0.46
malonylcarnitine	1.55	1.63	1.79	1.27
malonate	0.39	1.15	1.42	3.01
valerate	1.12	0.09	0.02	0.06
caproate (6:0)	1.08	0.90	0.31	0.68
heptanoate (7:0)	1.34	1.16	0.60	0.98
caprylate (8:0)	0.86	1.34	0.93	1.59
caprate (10:0)	1.29	4.27	1.43	0.53
laurate (12:0)	2.32	5.52	1.48	0.20
myristate (14:0)	2.89	2.28	0.85	0.10
myristoleate (14:1n5)	2.08	0.35	0.25	0.20
pentadecanoate (15:0)	2.82	0.11	0.07	0.07
palmitate (16:0)	1.76	1.29	0.73	0.32
palmitoleate (16:1n7)	1.38	0.33	0.21	0.18
margarate (17:0)	1.95	0.39	0.17	0.10
10-heptadecenoate (17:1n7)	1.18	0.18	0.10	0.11
stearate (18:0)	2.55	0.80	0.47	0.33
oleate/vaccenate (18:1)	1.36	0.51	0.31	0.26
nonadecanoate (19:0)	2.43	0.58	0.22	0.09
10-nonadecenoate (19:1n9)	1.26	1.29	0.23	0.18
trans-nonadecenoate (tr 19:1)*	4.65	0.04	0.36	0.10
arachidate (20:0)	4.04	0.70	0.29	0.15
eicosenoate (20:1)	2.47	1.16	0.48	0.18
behenate (22:0)*	4.12	0.59	0.25	0.18
erucate (22:1n9)	6.12	0.53	0.23	0.09
nervonate (24:1n9)*	5.84	0.49	0.27	0.06
heneicosapentaenoate (21:5n3)	0.25	1.29	0.56	0.90
hexadecadienoate (16:2n6)	1.26	0.27	0.16	0.26
stearidonate (18:4n3)	0.75	0.47	0.22	0.55
eicosapentaenoate (EPA; 20:5n3)	1.42	0.25	0.25	0.27
docosapentaenoate (n3 DPA; 22:5n3)	3.62	0.21	0.07	0.11
docosahexaenoate (DHA; 22:6n3)	1.36	0.30	0.24	0.25
linoleate (18:2n6)	0.63	0.85	0.52	0.78
linolenate [alpha or gamma; (18:3n3 or 6)]	0.34	1.18	0.60	1.11
dihomo-linolenate (20:3n3 or n6)	4.23	0.18	0.07	0.14
arachidonate (20:4n6)	1.93	0.27	0.24	0.26
adrenate (22:4n6)	4.27	0.31	0.12	0.08
docosapentaenoate (n6 DPA; 22:5n6)	3.84	0.09	0.06	0.07
docosadienoate (22:2n6)	12.87	0.62	0.21	0.03
dihomo-linoleate (20:2n6)	9.48	0.40	0.12	0.04
13-methylmyristate	5.86	0.00	0.00	0.00
17-methylstearate	3.53	0.78	0.36	0.08
glutarate (pentanedioate)	0.28	0.11	0.14	0.13
2-hydroxyglutarate	0.58	0.02	1.33	0.20
2-hydroxyadipate	0.71	0.14	0.36	0.31
maleate	1.12	1.02	1.25	1.19

suberate (octanedioate)	1.01	0.11	0.25	0.32
azelate (nonanedioate)	0.73	0.24	0.52	0.91
sebacate (decanedioate)	0.47	0.14	0.17	0.19
dodecanedioate	0.44	0.16	0.14	0.18
hexadecanedioate	1.20	0.06	0.18	0.13
octadecanedioate	0.21	0.61	0.33	0.78
1-dihomo-linoleoylglycerol (20:2)	3.25	0.26	0.13	0.17
butyrylcarnitine (C4)	0.58	0.31	0.21	0.56
propionylcarnitine (C3)	0.69	0.86	2.10	1.35
methylmalonate (MMA)	0.31	0.66	0.84	1.59
acetylcarnitine (C2)	1.28	1.20	1.33	2.12
3-hydroxybutyrylcarnitine (1)	1.98	1.01	1.66	2.43
3-hydroxybutyrylcarnitine (2)	1.24	0.41	0.40	0.91
hexanoylcarnitine (C6)	1.16	4.23	4.25	2.50
octanoylcarnitine (C8)	1.08	6.58	4.65	3.23
decanoylcarnitine (C10)	0.82	9.31	7.60	1.89
laurylcarnitine (C12)	1.24	12.69	8.67	1.26
myristoylcarnitine (C14)	1.69	4.19	3.89	0.42
palmitoylcarnitine (C16)	2.73	1.04	0.54	0.18
palmitoleoylcarnitine (C16:1)*	1.43	3.81	1.32	0.60
stearoylcarnitine (C18)	3.65	0.36	0.21	0.21
linoleoylcarnitine (C18:2)*	1.61	2.94	0.71	0.24
oleoylcarnitine (C18:1)	1.86	0.79	0.25	0.17
myristoleoylcarnitine (C14:1)*	1.44	4.86	2.56	1.28
adipoylcarnitine (C6-DC)	1.17	5.85	7.18	5.15
arachidoylcarnitine (C20)*	9.47	0.36	0.12	0.25
arachidonoylcarnitine (C20:4)	2.19	4.05	0.81	1.35
behenoylcarnitine (C22)*	3.10	0.53	0.47	0.39
dihomo-linoleoylcarnitine (C20:2)*	5.52	0.52	0.18	0.35
eicosenoylcarnitine (C20:1)*	3.97	0.25	0.16	0.23
lignoceroylcarnitine (C24)*	2.35	0.51	0.20	0.40
margaroylcarnitine*	3.74	0.20	0.11	0.10
deoxycarnitine	1.78	0.01	0.01	0.01
carnitine	1.24	0.92	1.73	2.27
3-hydroxybutyrate (BHBA)	0.76	0.78	1.69	1.33
palmitoylcholine	2.43	0.91	0.36	0.36
2-hydroxypalmitate	6.53	0.06	0.04	0.01
2-hydroxystearate	3.39	0.25	0.25	0.12
2-hydroxybehenate	4.61	0.57	0.47	0.30
3-hydroxyhexanoate	1.29	0.37	0.29	0.42
3-hydroxydecanoate	1.32	0.38	0.43	0.43
3-hydroxylaurate	1.33	0.87	0.74	1.14
13-HODE + 9-HODE	0.59	0.87	0.86	0.98
10-hydroxystearate	4.73	0.00	0.09	0.08
12,13-DiHOME	0.35	1.37	0.72	0.98
9,10-DiHOME	0.62	0.45	0.22	0.25
12(13)-EpOME	0.58	0.91	0.71	0.84
oleoyl ethanolamide	1.44	0.26	0.07	0.11
palmitoyl ethanolamide	1.98	0.18	0.09	0.09
stearoyl ethanolamide	2.93	0.16	0.09	0.05

linoleoyl ethanolamide	0.33	0.72	0.20	0.37
arachidoyl ethanolamide (20:0)*	3.03	0.17	0.17	0.17
lignoceroyl ethanolamide (24:0)*	10.33	0.13	0.12	0.12
N-palmitoylserine	2.44	0.02	0.01	0.01
myo-inositol	0.99	3.85	9.00	6.93
chiro-inositol	0.32	0.12	5.37	4.45
pinitol	0.32	2.34	7.47	7.85
choline	1.37	0.79	0.83	0.76
choline phosphate	0.58	0.00	0.03	0.02
glycerophosphorylcholine (GPC)	1.01	0.78	3.33	1.13
glycerophosphoethanolamine	0.91	0.31	0.49	0.46
glycerophosphoserine*	2.91	0.95	0.64	0.28
glycerophosphoinositol*	5.17	0.48	0.31	0.26
trimethylamine N-oxide	0.88	4.83	1.31	0.21
1,2-dipalmitoyl-GPC (16:0/16:0)	1.80	0.71	0.37	0.54
1-palmitoyl-2-palmitoleoyl-GPC (16:0/16:1)*	0.37	0.42	0.43	0.86
1-palmitoyl-2-stearoyl-GPC (16:0/18:0)	1.73	0.63	0.41	0.62
1-palmitoyl-2-oleoyl-GPC (16:0/18:1)	0.39	0.32	0.46	1.09
1-palmitoyl-2-linoleoyl-GPC (16:0/18:2)	0.43	0.55	0.72	1.25
1-palmitoyl-2-arachidonoyl-GPC (16:0/20:4n6)	0.66	0.38	1.01	1.08
1-stearoyl-2-oleoyl-GPC (18:0/18:1)	0.37	0.38	0.57	1.38
1-stearoyl-2-linoleoyl-GPC (18:0/18:2)*	0.32	0.92	1.29	1.73
1-oleoyl-2-linoleoyl-GPC (18:1/18:2)*	0.22	0.74	0.80	1.68
1,2-dilinoleoyl-GPC (18:2/18:2)	0.27	0.64	1.46	1.20
1-linoleoyl-2-linolenoyl-GPC (18:2/18:3)*	0.22	0.89	1.58	1.05
1-stearoyl-2-arachidonoyl-GPC (18:0/20:4)	0.60	0.50	1.65	1.24
1-linoleoyl-2-arachidonoyl-GPC (18:2/20:4n6)*	0.30	0.44	4.98	0.90
1,2-dipalmitoyl-GPE (16:0/16:0)*	1.51	0.03	0.61	0.48
1-palmitoyl-2-oleoyl-GPE (16:0/18:1)	0.93	0.05	1.45	0.30
1-palmitoyl-2-linoleoyl-GPE (16:0/18:2)	0.56	0.12	3.03	0.73
1-palmitoyl-2-arachidonoyl-GPE (16:0/20:4)*	1.23	0.14	0.65	0.51
1-stearoyl-2-oleoyl-GPE (18:0/18:1)	0.36	0.62	2.40	2.23
1-stearoyl-2-linoleoyl-GPE (18:0/18:2)*	0.45	1.07	4.22	2.01
1-oleoyl-2-linoleoyl-GPE (18:1/18:2)*	0.31	1.19	1.67	3.13
1,2-dilinoleoyl-GPE (18:2/18:2)*	0.26	1.34	3.48	3.88
1-stearoyl-2-arachidonoyl-GPE (18:0/20:4)	0.52	1.52	0.90	2.74
1-stearoyl-2-oleoyl-GPS (18:0/18:1)	0.85	0.46	0.31	1.40
1-palmitoyl-2-linoleoyl-GPI (16:0/18:2)	0.28	0.78	1.90	1.10
1-stearoyl-2-arachidonoyl-GPI (18:0/20:4)	0.53	0.63	0.26	1.25
1-palmitoyl-GPA (16:0)	0.56	0.07	0.07	0.06
1-stearoyl-GPA (18:0)	0.55	0.15	0.15	0.15
1-oleoyl-GPA (18:1)	0.47	0.18	0.18	0.18
1-palmitoyl-GPC (16:0)	0.30	0.82	0.82	0.92
2-palmitoyl-GPC (16:0)*	0.39	1.14	2.09	1.22
1-palmitoleoyl-GPC (16:1)*	0.28	1.83	0.87	0.93
1-stearoyl-GPC (18:0)	0.31	0.68	0.65	0.87
1-oleoyl-GPC (18:1)	0.17	0.74	0.64	1.06
1-linoleoyl-GPC (18:2)	0.11	1.35	1.62	1.40
1-arachidonoyl-GPC (20:4n6)*	0.31	0.40	3.26	0.07
1-lignoceroyl-GPC (24:0)	0.43	0.64	0.35	1.13

1-palmitoyl-GPE (16:0)	0.86	**0.18**	0.55	**0.36**
1-stearoyl-GPE (18:0)	**0.41**	1.11	0.92	**1.39**
2-stearoyl-GPE (18:0)*	0.70	0.71	0.59	0.91
1-oleoyl-GPE (18:1)	**0.35**	0.55	1.53	1.05
1-linoleoyl-GPE (18:2)*	**0.12**	0.54	**5.81**	**1.43**
1-arachidonoyl-GPE (20:4n6)*	0.73	0.52	**0.55**	1.29
1-stearoyl-GPS (18:0)*	0.86	1.14	**0.27**	0.96
1-palmitoyl-GPG (16:0)*	0.71	**0.11**	**0.30**	**0.14**
1-stearoyl-GPG (18:0)	0.81	**0.05**	0.42	0.29
1-oleoyl-GPG (18:1)*	0.44	**0.02**	**2.52**	**0.10**
1-linoleoyl-GPG (18:2)*	**0.08**	0.08	**9.43**	0.57
1-palmitoyl-GPI (16:0)	**0.21**	1.38	0.73	0.85
1-stearoyl-GPI (18:0)	1.07	0.83	**0.22**	0.67
galactosylglycerol*	0.43	**15.10**	0.51	0.44
digalactosylglycerol*	0.51	**10.23**	0.22	0.22
1-palmitoyl-2-linoleoyl-digalactosylglycerol (16:0/18:2)*	0.65	**0.39**	0.89	**3.21**
1-palmitoyl-2-linoleoyl-galactosylglycerol (16:0/18:2)*	1.15	**0.05**	**0.09**	0.88
1-(1-enyl-palmitoyl)-2-oleoyl-GPE (P-16:0/18:1)*	**0.38**	**0.16**	**3.01**	0.53
1-(1-enyl-palmitoyl)-2-linoleoyl-GPE (P-16:0/18:2)*	**0.27**	**0.11**	**1.93**	0.38
1-(1-enyl-palmitoyl)-2-palmitoyl-GPC (P-16:0/16:0)*	**2.92**	0.67	**0.17**	0.70
1-(1-enyl-palmitoyl)-2-arachidonoyl-GPE (P-16:0/20:4)*	0.74	0.43	**0.34**	1.59
1-(1-enyl-palmitoyl)-2-oleoyl-GPC (P-16:0/18:1)*	0.71	**0.41**	0.58	1.33
1-(1-enyl-stearoyl)-2-oleoyl-GPE (P-18:0/18:1)	0.51	**0.23**	**3.72**	1.03
1-(1-enyl-stearoyl)-2-linoleoyl-GPE (P-18:0/18:2)*	0.50	**0.31**	0.90	1.54
1-(1-enyl-palmitoyl)-2-arachidonoyl-GPC (P-16:0/20:4)*	1.08	0.68	0.61	**2.43**
1-(1-enyl-palmitoyl)-2-linoleoyl-GPC (P-16:0/18:2)*	0.74	**0.23**	**0.33**	1.08
1-(1-enyl-stearoyl)-2-arachidonoyl-GPE (P-18:0/20:4)*	0.79	0.40	0.47	1.91
1-(1-enyl-palmitoyl)-GPC (P-16:0)*	**6.83**	0.43	**0.11**	0.38
1-(1-enyl-palmitoyl)-GPE (P-16:0)*	1.11	**0.36**	**0.30**	**0.34**
1-(1-enyl-oleoyl)-GPE (P-18:1)*	1.67	**0.40**	**0.08**	0.44
1-(1-enyl-stearoyl)-GPE (P-18:0)*	**2.29**	**0.29**	**0.18**	**0.31**
1-(1-enyl-oleoyl)-2-linoleoyl-GPE (P-18:1/18:2)*	0.56	0.35	**0.36**	1.70
glycerol	0.79	**0.56**	0.75	**0.22**
glycerol 3-phosphate	1.99	**0.01**	**0.06**	**0.02**
glycerophosphoglycerol	0.79	**0.07**	1.37	**0.21**
1-myristoylglycerol (14:0)	0.91	**0.48**	**0.49**	**0.53**
1-pentadecanoylglycerol (15:0)	1.88	**0.02**	**0.02**	**0.02**
1-palmitoylglycerol (16:0)	1.02	**0.47**	**0.42**	0.57
1-palmitoleoylglycerol (16:1)*	0.72	**0.26**	**0.28**	**0.40**
1-margaroylglycerol (17:0)	0.84	**0.12**	**0.12**	**0.12**
1-oleoylglycerol (18:1)	0.54	**0.39**	**0.60**	0.65
1-linoleoylglycerol (18:2)	0.40	**0.48**	0.60	1.06
1-linolenoylglycerol (18:3)	**0.30**	0.58	0.74	1.16
1-dihomo-linolenylglycerol (20:3)	2.23	**0.25**	**0.13**	**0.29**
1-arachidonylglycerol (20:4)	1.83	0.63	**0.27**	0.77
1-eicosapentaenoylglycerol (20:5)*	0.57	0.98	**0.36**	1.62
1-docosahexaenoylglycerol (22:6)	0.97	**0.75**	**0.28**	0.88
2-myristoylglycerol (14:0)	0.55	1.29	1.09	0.91
2-palmitoylglycerol (16:0)	1.26	0.72	0.82	0.58
2-palmitoleoylglycerol (16:1)*	0.64	0.71	0.67	0.98

2-oleoylglycerol (18:1)	0.34	0.94	0.99	1.88
2-linoleoylglycerol (18:2)	0.32	1.18	0.76	2.06
2-arachidonoylglycerol (20:4)	1.22	1.30	0.59	1.08
2-docosahexaenoylglycerol (22:6)*	0.62	1.80	0.86	2.07
palmitoyl-linoleoyl-glycerol (16:0/18:2) [2]*	0.69	0.20	0.65	0.15
oleoyl-linoleoyl-glycerol (18:1/18:2) [1]	0.56	1.12	1.01	0.30
oleoyl-linoleoyl-glycerol (18:1/18:2) [2]	0.65	0.16	0.59	0.17
linoleoyl-linoleoyl-glycerol (18:2/18:2) [1]*	0.68	0.69	0.87	0.27
linoleoyl-linoleoyl-glycerol (18:2/18:2) [2]*	0.68	0.18	0.65	0.17
linoleoyl-linolenoyl-glycerol (18:2/18:3) [1]*	0.61	0.93	0.68	0.40
linoleoyl-linolenoyl-glycerol (18:2/18:3) [2]*	0.59	0.20	0.68	0.22
linoleoyl-arachidonoyl-glycerol (18:2/20:4) [2]*	1.23	0.41	0.41	0.63
linoleoyl-docosahexaenoyl-glycerol (18:2/22:6) [2]*	1.26	0.74	0.55	1.24
sphinganine	1.78	0.24	0.48	0.05
3-ketosphinganine	1.63	0.00	0.00	0.00
N-palmitoyl-sphinganine (d18:0/16:0)	3.23	0.68	0.20	0.39
N-palmitoyl-sphingadienine (d18:2/16:0)*	4.57	0.07	0.07	0.05
palmitoyl dihydrosphingomyelin (d18:0/16:0)*	3.45	0.35	0.17	0.24
behenoyl dihydrosphingomyelin (d18:0/22:0)*	2.41	0.57	0.25	0.31
palmitoyl sphingomyelin (d18:1/16:0)	2.58	0.29	0.26	0.36
stearoyl sphingomyelin (d18:1/18:0)	2.79	0.14	0.13	0.25
behenoyl sphingomyelin (d18:1/22:0)*	3.24	0.29	0.18	0.31
tricosanoyl sphingomyelin (d18:1/23:0)*	2.80	0.48	0.29	0.30
lignoceroyl sphingomyelin (d18:1/24:0)	3.52	0.53	0.27	0.27
sphingomyelin (d18:1/14:0, d16:1/16:0)*	2.97	0.27	0.21	0.26
sphingomyelin (d17:1/16:0, d18:1/15:0, d16:1/17:0)*	2.43	0.21	0.20	0.15
sphingomyelin (d18:2/16:0, d18:1/16:1)*	2.32	0.18	0.22	0.34
sphingomyelin (d18:1/17:0, d17:1/18:0, d19:1/16:0)	1.86	0.09	0.11	0.15
sphingomyelin (d18:1/20:0, d16:1/22:0)*	1.97	0.18	0.16	0.33
sphingomyelin (d18:1/24:1, d18:2/24:0)*	3.35	0.20	0.23	0.18
sphingosine	2.74	0.20	0.43	0.05
N-acetylsphingosine	4.91	0.02	0.19	0.02
phytosphingosine	0.43	2.27	9.68	0.71
sphingomyelin (d18:0/18:0, d19:0/17:0)*	3.44	0.26	0.10	0.12
heptadecasphingosine (d17:1)	2.20	0.27	0.32	0.05
hexadecasphingosine (d16:1)*	3.09	0.16	0.14	0.07
N-stearoyl-sphinganine (d18:0/18:0)*	3.06	0.31	0.35	0.09
hexadecasphinganine (d16:0)*	2.20	0.13	0.10	0.10
N-(2-hydroxypalmitoyl)-sphingosine (d18:1/16:0(2OH))	5.73	0.06	0.06	0.04
N-oleoyl-sphingosine (d18:1/18:1)*	15.33	0.17	0.03	0.01
N-palmitoyl-sphingosine (d18:1/16:0)	3.26	0.34	0.21	0.34
N-stearoyl-sphingosine (d18:1/18:0)*	9.06	0.22	0.25	0.08
ceramide (d18:1/14:0, d16:1/16:0)*	4.49	0.55	0.15	0.15
ceramide (d18:1/17:0, d17:1/18:0)*	3.50	0.18	0.13	0.11
ceramide (d18:1/20:0, d16:1/22:0, d20:1/18:0)*	4.06	0.16	0.25	0.14
ceramide (d18:2/24:1, d18:1/24:2)*	4.11	0.10	0.27	0.08
glycosyl-N-palmitoyl-sphingosine (d18:1/16:0)	4.77	0.25	0.05	0.20
lactosyl-N-palmitoyl-sphingosine (d18:1/16:0)	75.79	0.01	0.01	0.01
lactosyl-N-nervonoyl-sphingosine (d18:1/24:1)*	36.72	0.03	0.03	0.03
eicosanoylsphingosine (d20:1)*	1.92	0.24	0.40	0.11

3-hydroxy-3-methylglutarate	0.18	0.10	0.18	0.11
mevalonate	0.41	0.06	5.24	1.19
mevalonolactone	0.75	0.21	6.82	1.32
lanosterol	2.35	0.38	0.25	0.35
desmosterol	2.81	0.80	0.73	0.72
cholesterol	1.91	0.73	0.71	0.51
coprostanol	1.37	0.69	0.69	0.69
4-cholesten-3-one	1.30	0.73	0.50	1.01
beta-sitosterol	0.44	0.72	1.85	1.31
stigmasterol	0.36	0.46	1.41	1.31
campesterol	0.72	0.76	1.26	0.78
ergosterol	0.30	0.49	0.87	0.69
7-hydroxycholesterol (alpha or beta)	3.11	0.80	0.38	0.32
21-hydroxypregnanolone disulfate	0.70	2.34	1.79	3.24
5alpha-pregnan-3beta,20alpha-diol disulfate	0.33	1.04	0.60	0.44
5alpha-pregnan-3(alpha or beta),20beta-diol disulfate	1.45	0.82	0.42	1.12
cholate	0.31	0.00	0.97	0.09
glycocholate	0.19	0.89	0.87	0.87
taurocholate	0.00	26.74	4.85	23.52
chenodeoxycholate	0.12	0.04	0.93	0.11
taurochenodeoxycholate	0.01	16.79	2.82	22.60
beta-muricholate	0.39	0.00	0.17	0.00
alpha-muricholate	0.20	0.00	0.58	0.01
tauro-beta-muricholate	0.00	17.61	1.47	11.43
cholate sulfate	0.58	0.18	0.38	1.13
deoxycholate	0.60	0.00	0.00	0.00
6-beta-hydroxylithocholate	0.49	0.03	0.07	0.03
lithocholate	0.88	0.03	0.03	0.03
ursodeoxycholate	0.22	0.17	0.26	0.17
tauroursodeoxycholate	0.00	10.20	1.30	11.62
dehydrolithocholate	1.09	0.04	0.04	0.04
7,12-diketolithocholate	1.30	0.16	0.16	0.16
6-oxolithocholate	0.35	0.91	0.91	0.91
7-ketolithocholate	0.21	0.18	0.27	0.18
hyocholate	1.64	0.02	0.03	0.04
3-dehydrocholate	0.63	0.02	0.06	0.02
12-dehydrocholate	2.83	0.01	0.01	0.01
taurocholenate sulfate	0.66	22.80	18.35	6.72
7-ketodeoxycholate	1.04	0.00	0.01	0.00
3b-hydroxy-5-cholenoic acid	1.59	0.05	0.09	0.11
taurochenodeoxycholate sulfate	0.30	4.32	3.64	5.49
ursodeoxycholate sulfate (2)	1.70	0.09	0.21	0.20
ursocholate	0.53	0.09	0.24	0.69
inosine	0.41	1.09	0.47	0.29
hypoxanthine	1.25	0.02	0.39	0.18
xanthine	2.02	0.08	0.29	0.17
xanthosine	1.15	1.28	0.05	0.08
N1-methylinosine	0.46	42.58	1.00	1.00
2'-deoxyinosine	1.10	0.18	0.12	0.03
urate	1.87	1.98	2.44	2.25

allantoin	0.71	4.67	4.62	5.60
allantoic acid	0.42	3.75	10.10	27.68
adenosine 5'-monophosphate (AMP)	2.82	0.01	0.04	0.04
adenosine-2',3'-cyclic monophosphate	2.55	0.11	0.11	0.11
adenosine	1.17	0.13	0.08	0.22
adenine	0.89	0.04	0.11	0.10
1-methyladenine	1.07	0.03	1.75	1.51
N1-methyladenosine	0.21	2.86	0.41	0.65
N6-carbamoylthreonyladenosine	0.61	6.72	2.70	6.74
2'-deoxyadenosine 5'-monophosphate	0.51	0.02	0.03	0.02
2'-deoxyadenosine	0.88	0.06	0.02	0.04
N6-succinyladenosine	1.15	12.28	15.13	0.54
guanosine-2',3'-cyclic monophosphate	2.06	0.10	0.10	0.10
guanosine	0.31	2.70	0.54	0.20
guanine	0.62	0.05	0.40	0.15
1-methylguanine	1.03	0.30	1.97	1.59
7-methylguanine	1.74	0.45	0.76	0.59
N1-methylguanosine	0.65	13.80	0.56	0.99
N2,N2-dimethylguanosine	0.12	62.84	3.10	1.00
N2,N2-dimethylguanine	0.79	1.63	22.91	0.84
2'-deoxyguanosine	0.72	0.63	0.20	0.07
N-carbamoylaspartate	0.54	0.06	2.35	1.53
dihydroorotate	2.03	0.79	8.41	3.67
orotate	0.68	5.10	10.67	4.22
orotidine	0.54	53.02	53.95	0.71
uridine 5'-monophosphate (UMP)	1.32	0.20	0.20	0.20
uridine 3'-monophosphate (3'-UMP)	4.45	0.16	0.16	0.16
uridine-2',3'-cyclic monophosphate	4.65	0.01	0.02	0.07
uridine	0.91	0.38	0.64	0.18
uracil	1.80	0.01	0.15	0.09
pseudouridine	1.46	0.32	0.37	0.11
2'-O-methyluridine	0.73	1.23	2.53	3.07
5-methyluridine (ribothymidine)	0.99	0.02	0.17	0.02
2'-deoxyuridine	0.93	0.16	0.19	0.02
3-ureidopropionate	2.88	0.04	0.05	0.16
beta-alanine	1.41	0.16	0.25	0.29
N-acetyl-beta-alanine	1.25	0.15	0.56	1.59
cytidine 5'-monophosphate (5'-CMP)	1.52	0.02	0.07	0.06
cytidine 3'-monophosphate (3'-CMP)	6.02	0.00	0.00	0.00
cytidine	1.21	1.14	0.11	0.21
cytosine	2.56	0.02	1.09	0.15
5-methylcytosine	0.95	0.05	1.84	0.26
2'-deoxycytidine 5'-monophosphate	0.56	0.03	0.02	0.02
2'-deoxycytidine	1.26	1.83	0.12	0.08
2'-O-methylcytidine	1.46	2.19	1.33	0.34
5-methyl-2'-deoxycytidine	1.04	1.26	0.27	0.24
thymidine 5'-monophosphate	0.57	0.24	0.24	0.24
thymidine	0.86	0.85	0.27	0.06
thymine	1.59	0.01	0.16	0.08
5,6-dihydrothymine	0.73	2.37	5.61	4.19

methylphosphate	1.07	0.70	1.05	5.46
quinolinate	1.28	1.60	1.22	1.53
nicotinate	0.91	0.00	0.22	0.13
nicotinate ribonucleoside	0.55	0.20	0.98	0.52
nicotinamide	0.45	3.70	0.65	1.04
nicotinamide ribonucleotide (NMN)	3.09	0.34	1.80	5.29
nicotinamide riboside	1.27	1.26	1.48	0.55
1-methylnicotinamide	1.08	2.12	1.85	2.06
trigonelline (N'-methylnicotinate)	1.09	0.23	0.57	0.54
N1-Methyl-2-pyridone-5-carboxamide	0.64	4.51	3.64	3.50
N1-Methyl-4-pyridone-3-carboxamide	0.64	0.86	1.05	0.35
riboflavin (Vitamin B2)	2.23	0.02	0.08	0.07
pantothenate	1.16	0.03	0.13	0.07
pantethine	4.00	0.05	0.04	0.05
pantetheine	1.61	0.05	0.06	0.07
threonate	0.82	2.51	1.96	1.42
oxalate (ethanedioate)	0.88	0.75	0.41	1.08
gulonate*	0.35	17.93	47.35	2.36
alpha-tocopherol	1.18	0.60	0.56	0.87
alpha-tocopherol acetate	0.48	1.23	1.52	1.70
delta-tocopherol	0.52	0.71	0.85	1.32
alpha-tocotrienol	0.40	0.48	0.76	1.08
gamma-tocotrienol	0.34	0.64	0.85	0.86
gamma-CEHC	1.87	0.87	0.91	0.94
gamma-tocopherol/beta-tocopherol	0.64	0.55	0.61	1.12
biotin	3.30	0.26	0.26	0.26
biopterin	2.86	0.83	1.34	1.06
dihydrobiopterin	4.50	0.56	0.74	1.05
pterin	2.10	0.03	0.04	0.04
protoporphyrin IX	3.09	0.36	0.43	0.42
heme	1.44	0.68	0.41	0.49
bilirubin (Z,Z)	1.62	4.83	4.79	3.97
bilirubin (E,E)*	0.96	1.57	1.35	1.38
biliverdin	1.44	1.77	1.91	1.12
I-urobilinogen	5.59	0.02	0.02	0.02
D-urobilin	2.54	0.11	0.11	0.11
thiamin (Vitamin B1)	1.39	0.25	0.60	0.30
thiamin monophosphate	1.30	0.36	0.36	0.36
hydroxymethylpyrimidine	0.85	0.37	0.37	0.37
pyridoxine (Vitamin B6)	0.48	0.22	0.30	0.22
pyridoxamine	2.06	0.15	0.30	0.10
pyridoxamine phosphate	1.03	0.27	0.27	0.27
pyridoxal	1.45	0.07	0.12	0.14
pyridoxate	1.94	0.10	0.17	0.31
4-hydroxyhippurate	0.30	21.59	33.36	14.13
4-hydroxymandelate	0.78	2.57	7.21	7.03
benzoate	1.00	0.78	0.67	0.79
4-hydroxybenzoate	0.29	0.15	0.53	0.49
2,4,6-trihydroxybenzoate	0.40	0.54	1.17	1.24
catechol sulfate	0.98	0.56	0.16	0.52

p-cresol sulfate	2.21	0.33	0.33	0.33
3-(3-hydroxyphenyl)propionate sulfate	3.83	1.96	0.47	2.63
2-(4-hydroxyphenyl)propionate	0.59	0.01	0.00	0.01
3-(3-hydroxyphenyl)propionate	0.54	0.00	0.00	0.00
3-(4-hydroxyphenyl)propionate	0.17	0.09	0.20	0.50
3-phenylpropionate (hydrocinnamate)	0.57	0.02	0.02	0.02
1-methylurate	1.55	0.19	1.33	1.17
caffeic acid sulfate	0.38	1.96	5.39	7.21
erythrose	3.09	0.26	1.59	0.19
genistein	0.22	0.29	1.32	0.62
3-dehydroshikimate	1.59	0.12	0.10	0.32
diaminopimelate	0.76	0.03	0.11	0.03
1-kestose	0.04	311.11	26.63	5.03
1,1-kestotetraose	1.74	1.93	0.89	0.11
apigenin	0.31	0.49	0.83	1.08
apigenin 7-O(6-malonyl-beta-D-glucoside)	0.11	520.33	894.11	1029.72
vanillate	0.52	0.27	0.73	0.94
vanillin	0.63	1.01	0.61	1.40
2,3-dihydroxyisovalerate	0.79	0.04	0.26	1.10
2,8-quinolinediol	27.53	0.01	0.01	0.01
2-isopropylmalate	0.57	0.06	0.08	0.09
2-oxindole-3-acetate	0.32	0.05	0.14	0.25
3,5-dihydroxybenzoic acid	0.68	0.19	0.19	0.19
gluconate	0.24	16.77	10.63	0.39
afromosin	0.38	0.26	0.47	0.33
beta-cryptoxanthin	0.30	0.42	0.86	1.04
beta-guanidinopropanoate	1.68	0.02	0.03	0.05
ciliatine (2-aminoethylphosphonate)	0.56	0.49	0.49	0.58
daidzein	0.52	0.13	0.69	0.32
daidzin	0.08	3.02	2.61	1.76
dihydroferulic acid	0.23	0.08	0.70	2.09
enterolactone	0.58	0.30	0.30	0.30
equol	0.14	0.05	0.05	0.05
equol sulfate	1.30	1.11	1.11	2.73
ergothioneine	1.52	0.20	0.06	0.34
erythritol	0.33	0.38	5.13	4.22
ferulate	0.40	0.08	0.71	0.51
ferulic acid 4-sulfate	0.20	2.46	7.19	6.69
ferulylglycine (1)	0.66	1.38	3.93	3.17
ferulylglycine (2)	0.67	1.17	3.00	1.55
formononetin	0.34	0.18	0.23	0.40
galacturonate	0.31	0.03	0.79	0.21
glycitein	0.31	0.93	2.09	1.13
homostachydrine*	0.21	0.87	1.21	0.99
indolin-2-one	0.48	0.13	0.13	0.13
naringenin	0.20	0.50	1.82	1.18
naringenin 7-glucuronide	0.61	3.23	2.87	1.00
N-glycolylneuraminate	1.27	0.81	14.47	0.31
nicotianamine	0.21	2.59	6.87	4.96
oleanolate	0.46	0.03	0.03	0.03

pheophorbide A	**0.39**	1.00	2.55	**0.23**
quinate	**0.33**	**4.48**	**6.08**	0.18
sinapate	**0.21**	**0.13**	0.93	0.37
soyasaponin I	**0.39**	**4.54**	**4.71**	**7.46**
soyasaponin II	0.40	**4.23**	**3.38**	**6.96**
soyasaponin III	**0.35**	**4.76**	1.32	**7.48**
stachydrine	0.97	**0.14**	**0.41**	**0.27**
syringic acid	**0.25**	**0.24**	0.60	1.03
tyrosol	0.87	**0.26**	**0.33**	1.16
sucrose-6-phosphate	0.89	**0.08**	**0.08**	**0.10**
feruloylputrescine	0.87	1.51	1.99	**3.03**
pyrraline	**0.21**	0.68	1.31	1.26
daidzein sulfate (2)	0.30	**4.14**	**7.00**	**8.36**
daidzein sulfate (1)	0.34	**2.86**	**5.26**	**5.67**
2-keto-3-deoxy-gluconate	0.28	0.69	1.30	**0.25**
vanillin sulfate	0.45	3.52	1.89	**4.01**
carotene diol (1)	**0.46**	0.53	0.94	1.13
carotene diol (2)	**0.57**	**0.39**	0.63	0.82
carotene diol (3)	**0.39**	**0.40**	0.89	1.00
dihydrocaffeate	**0.46**	**0.01**	**0.01**	0.17
4-hydroxycinnamate	0.70	**0.31**	0.95	0.37
3-deoxyoctulosonate	0.57	0.76	2.14	**0.21**
vancomycin	1.00	1.00	**2.38**	1.00
metronidazole	1.00	1.00	1.00	**3.50**
amoxicillin	1.00	**4.27**	1.00	1.00
salicylate	**0.47**	**0.32**	0.56	**0.53**
1,3-propanediol	0.42	0.39	0.33	0.42
sulfate*	0.92	**0.52**	**2.29**	0.70
O-sulfo-L-tyrosine	0.61	2.43	**5.08**	**3.73**
1-(3-aminopropyl)-2-pyrrolidone	0.98	0.65	0.71	**0.26**
2-aminophenol sulfate	0.82	0.82	0.84	0.95
HEPES	1.00	1.79	1.00	16.35
succinimide	1.52	**0.33**	**0.67**	**0.27**
thioproline	1.34	**0.52**	1.44	1.03

Heat map of statistically significant biochemicals profiled in this study. Red and green shaded cells indicate p⩽0.05 (red indicates that the mean values are significantly higher for that comparison; green values significantly lower). Light red and light green shaded cells indicate 0.05<p<0.10 (light red indicates that the mean values trend higher for that comparison; light green values trend lower).
